# Age-related widening of sex differences in upper-body strength and double poling performance in U16-U23 cross-country skiers and biathletes

**DOI:** 10.3389/fphys.2026.1890471

**Published:** 2026-10-27

**Authors:** Carl-Maximilian Wagner, Øyvind Sandbakk, Stephan Schiemann, Daniel Röhrs, Tobias Schmidt, Michael Keiner

**Affiliations:** 1 Institute of Exercise, Sport and Health, Leuphana University Lüneburg, Lüneburg, Germany; 2 School of Sport Science, UiT The Arctic University of Norway, Tromsø, Norway; 3 Department of Physical Performance, Norwegian School of Sport Sciences, Oslo, Norway; 4 Department of Sports Medicine, Institute of Human Movement Science, University of Hamburg, Hamburg, Germany; 5 Department of Sport Science, Medical School Hamburg, Hamburg, Germany; 6 Department of Sport Science, German University of Health and Sport, Ismaning, Germany

**Keywords:** adolescent athletes, age-related changes, biathlon, cross-country skiing, double poling, sex differences, upper-body maximal strength

## Abstract

**Introduction:**

Sex differences in the performance of elite cross-country skiers are reported enlarged in techniques with a higher upper-body contribution, such as double poling (DP) and G3 skating. However, little is known about the development of upper-body strength, aerobic power, and DP performance throughout adolescence and early adulthood. Therefore, this study examined age- and sex-related differences in upper-body characteristics and their associations with DP performance.

**Methods:**

Ninety-eight competitive cross-country skiers and biathletes (47 males, 51 females) from the U16 to U23 age categories completed a 15-s DP ergometer sprint test (DPP_15s_), and an incremental DP ergometer test to volitional exhaustion (DPP_peak_). Maximal strength was assessed using one-repetition maximum tests in the bench press (1RM_press_) and bench pull (1RM_pull_). Maximal oxygen uptake (VO_2max_) was determined during treadmill running with poles. Age- and sex-related differences were analyzed using two-way MANOVAs followed by separate two-way ANOVAs. Partial Pearson correlations were used to examine associations between maximal strength, DP performance, and aerobic power while controlling for age and sex.

**Results:**

Males demonstrated higher values across all performance measures, which generally increased with age. Significant age × sex interactions were observed for absolute 1RM_pull_ (η²=0.13, p=0.010), 1RM_press_ (η²=0.12, p=0.010), DPP_peak_ (η²=0.14, p=0.003), VO_2peak_ during DP (η²=0.14, p=0.003) and VO_2max_ (η²=0.11, p=0.018), indicating larger absolute sex differences in older age groups. No significant age × sex interactions were observed for DPP_15s_ (η²=0.05, p=0.228) or body-mass normalized measures, except for VO_2peak_ during DP (η²=0.11, p=0.019). 1RM was moderately to strongly associated with DPP_15s_, DPP_peak_ and VO_2peak_ during DP (r =0.50–0.61), whereas associations were weaker after normalization to body mass (r =0.13–0.39). Aerobic power showed strong associations with DPP_peak_ (r=0.73–0.82).

**Conclusion:**

Absolute sex differences in upper-body performance increased across age-groups and were more pronounced than previously reported for lower-body performance. However, body-mass normalized differences were largely stable across age groups. Upper-body strength was consistently associated with DP performance after accounting for age and sex, highlighting its potential relevance for DP performance development in youth athletes. However, the cross-sectional design precludes attribution of these age-related differences to maturation, training, or other developmental factors.

## 1 Introduction

Sex-based performance differences in endurance sports, including cross-country skiing, are well documented (Sollie and Losnegard, 2022; Hunter et al., 2023; Solli et al., 2024; Wagner et al., 2025). In competitive settings, men typically outperform women by 10 – 15% in endurance performance (Solli et al., 2024; Talsnes et al., 2025), with laboratory-based tests showing greater disparities (up to 25%), particularly in whole-body or lower-body dominant endurance tasks (McGawley and Holmberg, 2014; Sollie and Losnegard, 2022; Wagner et al., 2025). These sex-differences emerge during early adolescence, coinciding with male puberty (Hunter et al., 2023), and remain stable across age categories from junior to senior elite levels (Sollie and Losnegard, 2022). The primary physiological driver of this gap in endurance is the higher peak oxygen uptake (VO_2peak_) rooted in higher hemoglobin levels observed in male athletes, which enhances aerobic energy delivery (McGawley and Holmberg, 2014; Hegge et al., 2016; Joyner, 2017; Sandbakk et al., 2018; Sollie and Losnegard, 2022; Hunter et al., 2023; Skattebo et al., 2025).

In cross-country skiing, performance differences between sexes become even more pronounced in techniques that emphasize upper-body contribution, such as double poling (DP) and G3 skating compared to lower-body dominant modes. For instance, elite male sprint skiers have been shown to produce 62–67% higher absolute power in poling-dominant techniques compared to females, exceeding the differences observed in lower-body dominant modes. Peak oxygen uptake during DP exhibited sex differences of 63%. Notably, although normalization to body mass or fat-free mass reduces these differences, substantial sex differences remain (Sandbakk et al., 2014). In DP ergometry, sex differences in maximal performance test can reach up to 87% with male athletes exhibiting 51 – 63% higher VO_2peak_ values (Hegge et al., 2016). Previous research concluded that these pronounced differences cannot be explained by differences in hemoglobin and body fat alone (Stefani, 2006; Calbet and Joyner, 2010; Sandbakk et al., 2014; Joyner, 2017).

Consequently, upper-body maximal strength may be an important contributor to cross-country skiing performance (Stoggl and Muller, 2009; Hegge et al., 2016), with predominantly moderate to strong associations reported between maximal strength and DP performance (r = 0.52 – 0.89; sex-adjusted: r = 0.50 – 0.82) (Osteras et al., 2016; Sunde et al., 2019; Wagner et al., 2025). In contrast, Stöggl et al. (2011) found no significant association between 1RM performance in the bench press and bench pull and peak DP velocity (r = 0.24 – 0.51). Such inconsistencies may reflect methodological differences in the assessment of DP performance, as well as the complexity of the skiing movement, in which performance depends not only on the capacity to produce force but also in its coordinated application within sport-specific techniques (Stöggl et al., 2011). Moreover, the reported associations were attenuated when maximal strength was normalized to fat-free mass (r = 0.35 – 0.49) or when other explanatory variables, such as lean mass and VO_2max_, were considered in multivariate models (Osteras et al., 2016; Wagner et al., 2025). This attenuation suggests that differences in fat-free mass may contribute to the relationship between absolute maximal strength and DP performance, as absolute 1RM performance reflects both morphological characteristics and neuromuscular force-generating capacity. More broadly, these findings suggest that DP performance likely reflects the interaction between the available muscle mass, its neuromuscular force-generation capacity, and the ability to sustain this force production through sufficient aerobic energy provision.

In this context, fractional utilization of VO_2max_ during DP has been reported to range between 85 and 95% (Stoggl et al., 2019). While earlier studies reported significant sex differences in this value (Sandbakk et al., 2014; Hegge et al., 2016), more recent studies found no significant differences between sexes (~89% in women and ~88% in men) (Losnegard, 2019; Hansen et al., 2021). It has been speculated that the lack of sex differences may reflect adaptations associated with increased upper-body specific training, particularly in female athletes (Walther et al., 2026), changes in DP technique, or equipment (e.g., poles) (Stoggl et al., 2019). However, differences between studies may also reflect methodological variations in testing protocols or measurement procedures. This development is highly relevant for cross-country skiing, as a greater proportional use of upper-body dominant sub-techniques, such as DP and G3, has been associated with superior performance in both male and female skiers (Strom Solli et al., 2020; Talsnes et al., 2025; Sandbakk et al., 2026). However, the extent to which these technical and training-related demands develop during adolescence and junior years remains insufficiently investigated.

Current research has predominantly focused on adult skiers, whereas evidence in adolescent and junior Nordic skiers remains limited, particularly regarding upper-body performance and associated sex differences (Sollie and Losnegard, 2022; Wagner et al., 2025). For instance, Sollie and Losnegard (2022) reported that sex differences in performance within age groups were comparable among elite adolescent, junior and world-class senior cross-country skiers. However, their analysis was restricted to G2 treadmill roller ski skating, a sub-technique with relatively limited reliance on upper-body poling, and performance was reported only relative to body mass. In another study of youth cross-country skiers, attenuated sex differences were reported for upper-body strength (19–36%, d= 1.25–1.47), DP power (31–33%, d= 1.20–1.62) and VO_2peak_ in DP (29%, d= 1.99). Although these findings contribute valuable insight into upper-body performance in youth skiers, they lack generalizability given the small samples size, narrow age range, and convenience sampling. Consequently, it remains unclear whether the substantially larger sex differences observed among senior skiers reflect a progressive developmental trajectory, potentially associated with maturation, or whether the attenuated differences reported in the study are specific to the sampled population. Thus, how sex differences in upper-body performance evolve across athlete development represents an important gap in the current literature. Addressing this gap is essential for optimizing training strategies tailored to the specific needs of young athletes. Understanding when and how sex-based differences in upper-body strength and endurance emerge can inform individualized training programs, particularly regarding the development of maximal strength in endurance athletes.

Therefore, the primary objective of this study was to characterize age- and sex-related differences in upper-body maximal strength, aerobic power, and DP ergometer performance among adolescent and junior cross-country skiers and biathletes. A secondary objective was to examine the associations between DP ergometer performance and upper-body strength independent of age and sex, in a larger population to minimize sample bias and increase statistical power, thereby improving generalizability of observed correlations compared with previous studies with smaller samples (Keiner et al., 2025). It was hypothesized that sex differences in absolute upper-body maximal strength, aerobic power, and DP ergometer performance would increase progressively across age categories, with the largest sex-related differences expected in the oldest age category. This was based on the premise that sex differences in physical performance emerge primarily during puberty and become more pronounced during post-puberty as sex-specific differences in body composition, muscle mass, and cardiorespiratory capacity develop. It was further hypothesized that these sex differences would be attenuated when performance was normalized to body mass. Finally, it was hypothesized that absolute upper-body maximal strength would be positively associated with DP ergometer performance, independent of age and sex.

## 2 Methods

### 2.1 Experimental design

To characterize age- and sex-related differences in upper-body endurance performance, a cross-sectional study was conducted. Participants completed a DP protocol consisting of submaximal 4-minute stages at fixed workload levels, a 15-second sprint-test, and an incremental test to the point of exhaustion for the determination peak oxygen uptake. On a separate testing day, upper body strength and maximal aerobic power were assessed to examine the extent to which these variables explained upper-body endurance performance after adjustment for age and sex (see **Figure 1**). All participants were familiar with the testing procedures through familiarization sessions and/or training experience. Verbal encouragement was provided throughout all tests to elicit maximal effort. To minimize potential confounding factors, participants were instructed to limit their training to a maximum of ninety minutes at low intensity (<75% of maximum heart rate) on the day preceding the testing. On testing days, participants refrained from engaging in any fatiguing exercise prior to the assessment. In addition, participants were asked to maintain their habitual dietary and hydration practices throughout the study period. Furthermore, participants were instructed to refrain from consuming large meals during the designated pre-testing period. In cases where participants regularly consumed caffeine, they were instructed to adhere to their usual daily intake while avoiding any additional caffeine consumption.

**Figure 1:**
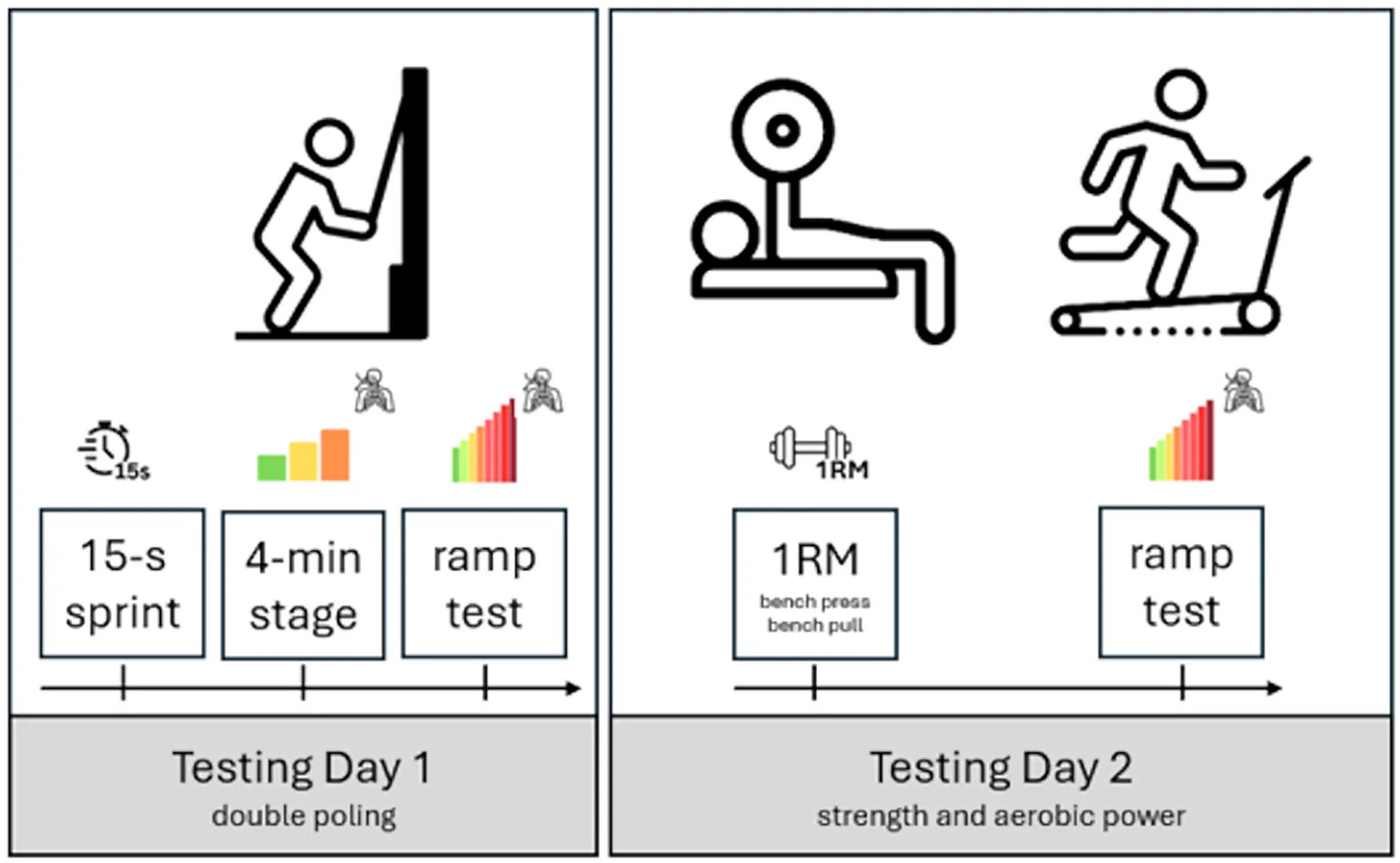
A schematic overview of the experimental design.

### 2.2 Participants

A total of 98 competitive skiers (male, n= 47; female, n= 51) voluntarily participated in this study. The cohort consisted of 47 cross-country skiers (male, n= 27; female, n= 20) and 51 biathletes (male, n= 20; female, n= 31) from regional elite training centers and teams, forming an *ad-hoc* sample of this study. Participants were categorized into four age groups according to chronological age (U16, 14–15 years; U18, 16–17 years; U20, 18–19 years; U23, 20–22 years). They were further classified into age-group specific performance tiers using a modified version of the athlete classification framework proposed by McKay, Stellingwerff (McKay et al., 2022). National level competitiveness (Tier 3) was defined as participation in at least three national-level races during the testing season, with ≥50% of finishes placed within the top half of the field. International-level athletes (Tier 4) were affiliated with the national federation’s age-group team structures and regularly competed in international events. Tier 5 represented a subset of Tier 4 athletes, who had achieved podium finishes at major international youth or junior events. Based on these performance indicators, the majority of participants (n= 68) met established criteria for elite-level status (Lorenz et al., 2013). In accordance with McKay, Stellingwerff (McKay et al., 2022), athlete classification was verified by cross-referencing competitive status from the preceding season and, when possible, the subsequent season. Accordingly, athletes were categorized as regional (Tier 2, n= 30, VO_2max_·BM^-1^ 62.3 ± 7.1 mL·kg^-1^·min^-1^), national (Tier 3, n = 49, VO_2max_·BM^-1^ 65.9 ± 7.6 mL·kg^-1^·min^-1^), international (Tier 4, n = 13, VO_2max_·BM^-1^ 70.3 ± 8.2 mL·kg^-1^·min^-1^), and Medalists (Tier 5, n= 6, VO_2max_·BM^-1^ 74.8 ± 9.2 mL·kg^-1^·min^-1^). All participants were engaged in structured training multiple times per week within provincial or national team structures and competed in at least national-level races. Mean training volume during the three months preceding data collection was 9.2 ± 2.5 (8.7 – 9.8) h·wk^−1^ (derived from participants’ training diaries cross-checked against coaches’ training plans). All participants reported prior engagement in structured resistance training, ranging from six months to several years, as well as regular core stability training, incorporating band or sling exercises, Swiss Ball routines, and various mat exercises, targeting lower back and abdominal muscles. All participants and their legal guardians (if the subjects were younger than 18 years) were informed about the aims of the study and the experimental risks involved with the research before providing written informed consent. Participants were informed that the participation was voluntary and that they could withdraw from the study at any time without consequences. Individuals who declined participation or withdrew were permitted to continue their regular training without any disadvantage. None of the participants reported any injuries at the time of testing. The study was performed in accordance with the Declaration of Helsinki and was approved by the Ethics Committee of the German University of Health and Sport (DHGS-EK-2023-004).

No a *priori* power analysis was performed because recruitment was constrained by the number of eligible athletes available for testing. As a reference, a power calculation based on the smallest sex-related effect reported in a comparable youth cohort (d = 1.20; Wagner et al., 2025) indicated that approximately 12 participants per group would be required to detect such an effect with 80% power at α = 0.05. Across four age categories and both sexes, this corresponds to an overall sample of approximately 96 participants, which is comparable to the present sample (n = 98). However, because subgroup sizes were unequal, some age × sex subgroups were below this benchmark.

### 2.3 Assessment of upper body maximal strength

Upper-body maximal strength was assessed using one-repetition maximum (1RM) testing in bench press and bench pull, following National Strength and Conditioning Association guidelines (Haff and Triplett, 2016) and previously described procedures (Akca, 2014; Schoenfeld et al., 2016). After a 10-min cycle-ergometer warm-up (<75% HR_max_), participants performed three sets of 2–5 repetitions at approximately 50 – 80% of 1RM. Subsequent attempts were initiated at approximately 90 – 95% of the estimated 1RM, with ≥5 min rest between attempts. Following each successful attempt, the load was increased by 2 – 5% until participants could not complete loads with proper technique (for a maximum of 2 failed attempts in a row). In the bench press, valid attempts required full elbow extension during the concentric phase after the barbell gently contacted the chest, with the head, upper back, and buttocks maintained in contact with the bench and the feet flat on the floor. For the prone bench pull, participants started with the arms fully extended, while the chest and lower extremities remained in contact with the bench. A successful attempt required the barbell to touch the bench with an elbow angle ≤90° (Akca, 2014; Schoenfeld et al., 2016). Testing was supervised by the same investigator and conducted on the same equipment. The highest successfully lifted load was recorded as the 1RM (bench press: 4 ± 1 attempts; bench pull: 3 ± 1 attempts). The reliability of assessments has been established, with intraclass correlation coefficients (ICC) ranging between 0.94-1.00 and 0.97-0.99 for bench press and bench pull, respectively (García-Ramos et al., 2019; Grgic et al., 2020; Sigvaldsen et al., 2023).

### 2.4 Assessment of maximal aerobic power

Maximal oxygen uptake was assessed using an incremental treadmill protocol using poles, following established procedures described previously (Wagner et al., 2024; Wagner et al., 2025). After a standardized 10-minute low-intensity warm-up (<75% HR_max_), participants completed an incremental running test to volitional exhaustion. The protocol commenced with a 4-minute initial stage at a velocity of 6 km·h^-^¹ and an incline of 5%. Thereafter, the inclination was set at 10.5%, and the velocity of the treadmill was augmented by 1 km·h^-^¹ every 60 seconds until exhaustion. The duration of the tests ranged from 4:28 to 10:05 minutes. Pulmonary gas exchange variables (VO_2_, VCO_2_) were continuously measured using a portable metabolic system (K5, COSMED, Rome, Italy) with a 10-second sampling interval. The K5 has demonstrated excellent intra- and inter-system reliability (ICCs ≥0.99) (Guidetti et al., 2018; Crouter et al., 2019) and has been validated against metabolic simulators (VacuMed, USA) yielding coefficients of determination of R^2^ ≥ 0.99. In addition, its accuracy has been confirmed against the Douglas bag method (Crouter et al., 2019). VO_2max_ was defined as the highest 30-second consecutive average of VO_2_. Heart rate was monitored throughout the test using a chest strap (Polar H10, Polar Electro Oy, Kempele, Finland). Peak blood lactate concentration ([La^-^]_peak_) was determined via capillary blood sampling 1.5 minutes post-exercise (EKF Biosen C-line analyzer, Barleben, Germany). VO_2max_ was confirmed if at least two of the following criteria were met: (1) VO_2_ plateau (<150 mL·min^-^¹ increase) (Brink-Elfegoun et al., 2007), (2) RER >1.10, (3) HR ≥95% of predicted or previously recorded HR_max_, (4) [La^-^]_peak_ >8.0 mmol·L^-^¹ (Sandbakk et al., 2010). All calibration procedures were conducted in accordance with the manufacturer’s guidelines, including volume calibration with a 3-L syringe and gas analyzer calibration using certified reference gases. The same spiroergometric system was used for both the VO_2max_ and DP tests to ensure methodological consistency.

### 2.5 Assessment of double poling performance

Upper-body endurance performance was assessed using a specially designed DP ergometer (SkiErg, Concept 2, Morrisville, VT) in accordance with established protocols (Wagner et al., 2024; Wagner et al., 2025). Participants adopted a position that closely resembled their habitual DP technique on skis and were allowed to self-select cadence throughout the testing session. Power output (stroke by stroke) was recorded using the integrated performance monitor (PM5, Concept 2, Morrisville, VT). To account for mass-dependent differences in force production and to standardize external loading across athletes, the flywheel deceleration rate (drag factor) was individually adjusted to 100% of each participant’s body mass via the damper settings (Faiss et al., 2015). The DP ergometer has demonstrated excellent reliability (ICC = 0.98 – 0.99) for both 20-sec sprint and 1000-m time-trial performances (Bucher et al., 2023; O'Brien et al., 2025). Moreover, the mean power output during 1000-m time-trial is strongly associated with on-snow skiing velocity (concordance correlation coefficient [r_c_] = 0.72; r^2^ = 0.78 – 0.87) (Bortolan et al., 2008; O'Brien et al., 2025).

#### 2.5.1 Maximal double poling power

Following a 4-minute warm-up at 60 W for women and 75 W for men on the ski ergometer, participants rested passively for 1 minute. Subsequently, the participants performed a 15-second sprint with maximal effort to determine their maximal double poling power (DPP_15s_), which was defined as the average 15-s power output. Participants were granted autonomy in initiating the sprint, while the endpoint was signaled verbally using a standardized countdown.

#### 2.5.2 Peak oxygen uptake test during double poling

After a 10-minute passive recovery, participants were equipped with a portable metabolic measurement system and completed three submaximal 4-min workloads at predetermined intensities. The initial workload was set at 72 W for women and 90 W for men, followed by incremental increases of 12 W and 15 W, respectively. The submaximal phase was terminated prematurely if the RER exceeded 1.0, indicating a substantial anaerobic contribution. After a subsequent 1-minute passive recovery, participants performed an incremental test to volitional exhaustion to determine VO_2peak_ DP. The test commenced at 90 to 120 W for women and 120 to 140 W for men, with the initial intensity determined by the number of submaximal stages completed. Work rate was increased continuously by 15 W·min^-1^ in women and 20 W·min^-1^ in men. Exhaustion was defined as a sustained reduction in power output of ≥5 W below the target workload for five consecutive strokes. Time to exhaustion ranged from 3:07 to 12:00 minutes across participants. The peak DP power output (DPP_PEAK_) was calculated using a linear interpolation method commonly applied in cycle ergometry (Kuipers et al., 1985):


DPPPEAK=DPPCOM+(t/60)•ΔW

Here, DPP_COM_ denotes the power output of the final stage that has been fully completed. The variable t represents the number of seconds that have elapsed in the final incomplete stage, and ΔW signifies the increase in power output for that stage (Alsobrook and Heil, 2009). VO_2peak_ DP was defined as the highest 30-second consecutive measured average of VO_2_.

### 2.6 Statistical analysis

All statistical analyses and data visualizations were conducted using R 4.4.3 (R Foundation for Statistical Computing, Vienna, Austria). Descriptive statistics are reported as mean ± standard deviation (SD), as well as median and interquartile range within box plots. For all performance variables, the best individual values were used in the analysis. The significance level was set *a priori* at p < 0.05 for all statistical tests. Normality of the dependent variables was examined using the Shapiro–Wilk test for the complete sample and separately for each age × sex subgroup. In addition, graphical diagnostics (Q-Q plots and histograms) were inspected to complement the inferential tests. Homogeneity of covariances was assessed using Box’s test, and homogeneity of variances was evaluated using Levene’s test and F_max_ statistics.

As the sample consisted of athletes from cross-country skiing and biathlon, and athletes were classified by the authors into performance tiers, two preliminary one-way multivariate analyses of variance (MANOVA) were performed to examine whether discipline (cross-country skiing, biathlon) or performance level (Tier 2 – 5) had a systematic impact on the performance variables. Significant multivariate effects were followed by univariate analyses of variance (ANOVA). Additionally, Chi^2^ (χ^2^) tests with Cramer V were used to evaluate potential imbalances in the distribution of sex and age across discipline and performance level categories, ensuring that observed effects were not attributable to uneven subgroup composition. Following Cohen (2013), small effects of Cramer’s V were defined as values below 0.30 for the association between age and discipline/performance level (df = 1) and below 0.21 for the associations between age and discipline/performance level (df = 3). The decision to pool groups in the main analysis was based on statistical results, practical considerations (i.e., similar training structures), and cell size requirements for factorial models.

Pairwise effect sizes (Hedges g) were calculated for all biologically meaningful contrasts: (1) sex differences within age groups, and (2) age-group differences independent of sex. The interpretation followed conventional benchmarks: small (g > 0.20), medium (g > 0.50), and large (g > 0.80) (Cohen, 2013).

The main analysis consisted of a series of two-way MANOVAs with age group (U16, U18, U20, U23) and sex (male, female) as fixed factors. MANOVAs were conducted, corresponding to the conceptual domains of dependent variables: (1) anthropometric characteristics, (2) maximal upper-body strength measures, (3) upper-body endurance performance, and (4) maximal aerobic power. In the presence of violations of multivariate normality or homogeneity of covariance matrices, Pillai’s trace was used as a robust multivariate test statistic. When a MANOVA indicated significant multivariate effects, separate two-way ANOVAs (age group • sex) were conducted for each dependent variable in the domain. Partial eta squared (η²_p_) was calculated as the measure of effect size. Interpretation thresholds followed established guidelines: small (0.01–0.06), medium (0.07–0.13), and large (≥ 0.14) (Cohen, 2013). For pairwise *post hoc* comparisons, p-values were adjusted using the Bonferroni correction to control the family-wise Type I error rate. When the assumption of homogeneity of variances was violated, Welch’s ANOVA and Welch’s t-tests were additionally computed as robust procedures to assess the robustness of the results. As the Welch analyses yielded the same significance patterns, the standard two-way ANOVAs were retained as the primary analysis framework in order to model the full age group × sex design and provide interpretable effect sizes. For non-significant contrasts in the ANOVA and *post hoc* analyses, two one-sided tests (TOST) of equivalence were conducted. Equivalence was evaluated on the standardized mean difference (Hedges’ g), using *a priori* defined bounds of ± 0.50 SD, which reflect a commonly used smallest worthwhile change in sport science based on Cohen’s conventions for small effects (Hopkins, 2004).

For the correlation analyses, bivariate one-tailed Pearson correlation analyses (Pearson's r) were conducted to examine the relationships between maximal strength, DP power, and aerobic power. Partial correlation coefficients were calculated to account for potential confounding effects of age and sex. Correlation strengths were interpreted according to the following thresholds: ≤ 0.1 = trivial, > 0.1–0.3 = small, > 0.3–0.5 = moderate, and > 0.5 = large (Cohen, 2013).

## 3 Results

### 3.1 Analyses of age group and sex differences

Significant main effects of age group and sex were observed across all measured domains, whereas age group × sex interactions were domain specific. Across domains, main effects were classified large according to predefined thresholds, with age effects ranging from η²_p_ = 0.23 to 0.29, compared with substantially larger sex effects ranging from η²_p_ = 0.67 to 0.72. Results of the follow-up ANOVA with full statistical details and group comparisons are summarized in **Table 1**. Descriptive statistics and pairwise effect sizes are summarized in **Table 2** and illustrated in **Figure 2**.

**Table 1:** Descriptive statistics (mean ± SD [CI]) for anthropometric, performance, and physiological variables across age groups and sex.

Variable	Sex	U16	Sex	U18	Sex	U20	Sex	U23
Anthropometrics								
Size (m)	♀ (n=16)	1.64 ± 0.06 (1.61 - 1.67)	♀ (n=20)	1.66 ± 0.06 (1.63 - 1.68)	♀ (n=6)	1.65 ± 0.04 (1.61 - 1.69)	♀ (n=9)	1.71 ± 0.03 (1.68 - 1.73)
	♂ (n=6)	1.7 ± 0.08 (1.7 - 1.85)	♂ (n=16)	1.79 ± 0.05 (1.77 - 1.81)	♂ (n=15)	1.85 ± 0.05 (1.82 - 1.87)	♂ (n=10)	1.82 ± 0.04 (1.80 - 1.85)
Weight (kg)	♀ (n=16)	52.95 ± 6.96 (49.24 - 56.66)	♀ (n=20)	57.15 ± 6.70 (54.01 - 60.29)	♀ (n=6)	60.45 ± 6.26 (53.88 - 67.02)	♀ (n=9)	62.40 ± 4.60 (58.86 - 65.94)
	♂ (n=6)	59.92 ± 7.20 (52.36 - 67.48)	♂ (n=16)	65.61 ± 7.54 (61.59 - 69.62)	♂ (n=15)	74.65 ± 4.91 (71.94 - 77.37)	♂ (n=10)	74.53 ± 4.41 (71.38 - 77.68)
Strength tests (one-repetition maximum)								
1RM bench pull (kg)	♀ (n=16)	40.31 ± 3.97 (38.20 - 42.43)	♀ (n=20)	48.75 ± 7.54 (45.22 - 52.28)	♀ (n=6)	52.92 ± 8.58 (43.92 - 61.92)	♀ (n=9)	59.17 ± 5.45 (54.98 - 63.35)
	♂ (n=6)	55.00 ± 7.25 (47.40 - 62.60)	♂ (n=16)	68.13 ± 8.68 (63.50 - 72.75)	♂ (n=15)	82.50 ± 10.09 (76.91 - 88.09)	♂ (n=10)	87.75 ± 6.82 (82.87 ± 92.63)
1RM·BM^−1^ bench pull (kg·kg^−1^)	♀ (n=16)	0.77 ± 0.07 (0.73 - 0.80)	♀ (n=20)	0.85 ± 0.10 (0.81 - 0.90)	♀ (n=6)	0.87 ± 0.09 (0.77 - 0.97)	♀ (n=9)	0.95 ± 0.11 (0.87 - 1.04)
	♂ (n=6)	0.92 ± 0.11 (0.81 - 1.04)	♂ (n=16)	1.04 ± 0.09 (0.99 - 1.09)	♂ (n=15)	1.11 ± 0.13 (1.04 - 1.18)	♂ (n=10)	1.18 ± 0.12 (1.10 - 1.27)
1RM bench press (kg)	♀ (n=16)	33.25 ± 4.63 (30.78 - 35.72)	♀ (n=20)	40.6 ± 6.6 (37.51 - 43.69)	♀ (n=6)	43.33 ± 9.31 (33.56 - 53.10)	♀ (n=9)	50.28 ± 3.84 (47.32 - 53.23)
	♂ (n=6)	48.33 ± 9.1 7 (38.71 - 57.96)	♂ (n=16)	63.59 ± 12.35 (57.01 - 70.17)	♂ (n=15)	75.33 ± 9.06 (70.32 - 80.35)	♂ (n=10)	81.75 ± 9.51 (74.95 - 88.55)
1RM·BM^−1^ bench press (kg·kg^−1^)	♀ (n=16)	0.63 ± 0.07 (0.59 - 0.67)	♀ (n=20)	0.71 ± 0.10 (0.67 - 0.76)	♀ (n=6)	0.72 ± 0.14 (0.57 - 0.87)	♀ (n=9)	0.81 ± 0.07 (0.75 - 0.86)
	♂ (n=6)	0.81 ± 0.13 (0.68 - 0.94)	♂ (n=16)	0.97 ± 0.14 (0.90 - 1.04)	♂ (n=15)	1.01 ± 0.11 (0.95 - 1.07)	♂ (n=10)	1.10 ± 0.16 (0.99 - 1.22)
Upper‐body power and capacity (double poling ergometry)								
DPP_peak_ (W)	♀ (n=16)	122.3 ± 14.8 (114.4 - 130.2)	♀ (n=20)	150.4 ± 24.6 (138.8 - 161.9)	♀ (n=6)	145.5 ± 24.2 (120.1 - 170.9)	♀ (n=9)	186.9 ± 19.9 (171.6 - 202.2)
	♂ (n=6)	188.5 ± 41.0 (145.5 - 231.5)	♂ (n=16)	216.1 ± 38.4 (195.7 - 236.6)	♂ (n=15)	264.6 ± 31.4 (247.2 - 282.0)	♂ (n=10)	289.9 ± 24.4 (272.5 - 307.3)
DPP_peak_·BM^−1^ (W·kg^−1^)	♀ (n=16)	2.3 ± 0.2 (2.2 - 2.4)	♀ (n=20)	2.6 ± 0.3 (2.5 - 2.8)	♀ (n=6)	2.4 ± 0.3 (2.1 - 2.8)	♀ (n=9)	3.0 ± 0.3 (2.8 - 3.2)
	♂ (n=6)	3.1 ± 0.4 (2.7 - 3.6)	♂ (n=16)	3.3 ± 0.4 (3.1 - 3.5)	♂ (n=15)	3.5 ± 0.4 (3.3 - 3.8)	♂ (n=10)	3.9 ± 0.3 (3.7 - 4.1)
VO_2peak_‐DP (mL·min^−1^)	♀ (n=16)	2581 ± 289 (2421 - 2741)	♀ (n=20)	3028 ± 414 (2834 - 3222)	♀ (n=6)	2837 ± 421 (2395 - 3280)	♀ (n=9)	3360 ± 272 (3150 - 3569)
	♂ (n=6)	3686 ± 629 (3026 - 4346)	♂ (n=16)	3918 ± 667 (3548 - 4287)	♂ (n=15)	4659 ± 404 (4436 - 4883)	♂ (n=10)	4853 ± 367 (4590 - 5115)
VO_2peak_‐DP·BM^−1^ (mL·kg^−1^ min^−1^)	♀ (n=16)	49.1 ± 5.2 (46.3 - 52.0)	♀ (n=20)	52.9 ± 4.3 (50.9 - 55.0)	♀ (n=6)	47.0 ± 5.8 (40.9 - 53.1)	♀ (n=9)	54.0 ± 4.6 (50.4 - 57.6)
	♂ (n=6)	61.3 ± 4.5 (56.5 - 66.0)	♂ (n=16)	59.5 ± 6.4 (56.0 - 63.0)	♂ (n=15)	63.2 ± 5.6 (60.0 - 66.3)	♂ (n=10)	65.2 ± 4.4 (62.0 - 68.3)
DPP_15s_ (W)	♀ (n=16)	149.9 ± 26.5 (135.8 - 164.1)	♀ (n=20)	195.5 ± 39.2 (177.2 - 213.8)	♀ (n=6)	226.8 ± 39.6 (185.2 - 268.4)	♀ (n=9)	249.6 ± 23.8 (231.3 - 267.8)
	♂ (n=6)	257.3 ± 54.7 (199.9 - 314.7)	♂ (n=16)	325.5 ± 80.7 (282.5 - 368.5)	♂ (n=15)	407.9 ± 71.4 (368.3 - 447.4)	♂ (n=10)	408.7 ± 66.4 (361.2 - 456.2)
DPP_15s_ ·BM^−1^ (W·kg^−1^)	♀ (n=16)	2.8 ± 0.4 (2.6 - 3.1)	♀ (n=20)	3.4 ± 0.5 (3.2 - 3.7)	♀ (n=6)	3.8 ± 0.8 (3.0 - 4.6)	♀ (n=9)	4.0 ± 0.4 (3.7 - 4.3)
	♂ (n=6)	4.3 ± 0.5 (3.8 - 4.8)	♂ (n=16)	4.9 ± 0.9 (4.4 - 5.4)	♂ (n=15)	5.5 ± 0.8 (5.0 - 5.9)	♂ (n=10)	5.5 ± 0.8 (4.9 - 6.1)
Maximal aerobic power and capacity (treadmill running with poles)								
VO_2max_ (mL·min^−1^)	♀ (n=16)	3075 ± 413 (2826 - 3325)	♀ (n=20)	3442 ± 409 (3239 - 3645)	♀ (n=6)	3430 ± 275 (3141 - 3718)	♀ (n=9)	3882 ± 349 (3613 - 4150)
	♂ (n=6)	4230 ± 872 (3146 - 5313)	♂ (n=16)	4584 ± 677 (4223 - 4944)	♂ (n=15)	5311 ± 353 (5115 - 5507)	♂ (n=10)	5652 ± 238 (5482 - 5822)
VO_2max_·BM^−1^ (mL·kg^−1^· min^−1^)	♀ (n=16)	59.8 ± 3.1 (57.9 - 61.7)	♀ (n=20)	60.6 ± 4.9 (58.0 - 63.1)	♀ (n=6)	57.0 ± 4.9 (51.9 - 62.2)	♀ (n=9)	62.3 ± 4.8 (58.6 - 66.0)
	♂ (n=6)	68.1 ± 11.3 (54.0 - 82.1)	♂ (n=16)	69.9 ± 6.9 (66.2 - 73.5)	♂ (n=15)	71.9 ± 6.6 (68.2 - 75.6)	♂ (n=10)	76.0 ± 4.2 (73.0 - 79.0)

1RM, one‐repetition maximum; BM, body mass; DPP_15s_, mean power during 15s double poling ergometer sprint; DPP_PEAK_, peak power during the double poling ergometry incremental test to exhaustion; VO_2max_, maximal oxygen uptake; VO_2peak_ DP, peak oxygen uptake during double poling ergometry; BM, body mass. Orange shading represents female athletes, and blue shading represents male athletes.

**Table 2:** Effect sizes (Hedges’ g), results of ANOVA and *post hoc* analyses for anthropometric, performance, and physiological variables across age groups and sex.

Variable	Effect (g) m/w U16	Effect (g) m/w U18	Effect (g) m/w U20	Effect (g) m/w U23	Effect (g) U16 vs U18	Effect (g) U16 vs U20	Effect (g) U16 vs U23	Effect (g) U18 vs U20	Effect (g) U18 vs U23	Effect (g) U20 vs U23	ANOVA Age (F/p/ηp²)	ANOVA Sex (F/p/ηp²)	ANOVA Interaction (F/p/ηp²)
Anthropometrics													
Size (m)	2.07† [0.92, 3.21]	2.48† [1.60, 3.37]	4.21† [2.59, 5.84]	3.21† [1.81, 4.61]	-0.47 [-1.01, 0.07]	-1.21* [-1.87, -0.55]	-1.13* [-1.80, -0.46]	-0.82* [-1.38, -0.26]	-0.63 [-1.20, -0.06]	0.28 [-0.35, 0.91]	F= 22.84 p<0.001 ηp²= 0.43	F= 174.36 p<0.001 ηp²= 0.66	F= 2.07 p= 0.109 ηp²= 0.06
Weight (kg)	0.95† [-0.05, 1.96]	1.17† [0.45, 1.89]	2.57† [1.32, 3.83]	2.57† [1.32, 3.82]	-0.75* [-1.30, -0.20]	-1.94* [-2.68, -1.21]	-1.80* [-2.54, -1.07]	-1.16* [-1.74, -0.58]	-0.97* [-1.56, -0.38]	0.22 [-0.41, 0.85]	F= 29.12 p<0.001 ηp²= 0.49	F= 56.76 p<0.001 ηp²= 0.39	F= 1.33 p= 0.269 ηp²= 0.04
Strength tests (one-repetition maximum)													
1RM bench pull (kg)	2.83* [1.54, 4.12]	2.35* [1.48, 3.21]	2.92* [1.60, 4.25]	4.40* [2.69, 6.11]	-1.15† [-1.73, -0.58]	-2.24† [-3.01, -1.47]	-2.37† [-3.18, -1.56]	-1.16† [-1.74, -0.58]	-1.20† [-1.81, -0.60]	-0.01† [-0.64, 0.62]	F= 78.40 p<0.001 ηp²= 0.72	F= 194.61 p<0.001 ηp²= 0.68	F= 4.32 p= 0.01 ηp²= 0.13
1RM·BM^−1^ bench pull (kg·kg^−1^)	1.84† [0.73, 2.95]	1.99† [1.18, 2.81]	1.87† [0.74, 3.00]	1.91† [0.79, 3.02]	-1.03* [-1.59, -0.46]	-1.68* [-2.39, -0.98]	-1.93* [-2.68, -1.17]	-0.72* [-1.28, -0.16]	-0.95* [-1.54, -0.36]	-0.20 [-0.83, 0.43]	F= 29.39 p<0.001 ηp²= 0.49	F= 86.35 p<0.001 ηp²= 0.49	F= 0.65 p= 0.587 ηp²= 0.02
1RM bench press (kg)	2.38* [1.18, 3.58]	2.35* [1.49, 3.21]	3.37* [1.94, 4.79]	4.06* [2.44, 5.68]	-1.02† [-1.58, -0.45]	-2.06† [-2.81, -1.32]	-2.10† [-2.88, -1.33]	-0.96† [-1.53, -0.39]	-0.99† [-1.58, -0.40]	-0.04† [-0.66, 0.59]	F= 59.84 p<0.001 ηp²= 0.67	F= 196.43 p<0.001 ηp²= 0.69	F= 4.11 p= 0.01 ηp²= 0.12
1RM·BM^−1^ bench press (kg·kg^−1^)	1.90† [0.78, 3.01]	2.17† [1.33, 3.01]	2.34† [1.13, 3.56]	2.22† [1.04, 3.39]	-0.93* [-1.49, -0.37]	-1.61* [-2.30, -0.91]	-1.75* [-2.48, -1.02]	-0.57 [-1.13, -0.02]	-0.75* [-1.33, -0.17]	-0.19 [-0.82, 0.44]	F= 26.31 p<0.001 ηp²= 0.47	F= 113.11 p<0.001 ηp²= 0.56	F= 0.98 p= 0.404 ηp²= 0.03
Upper‐body power and capacity (double poling ergometry)													
DPP_peak_ (W)	2.63* [1.39, 3.88]	2.04* [1.22, 2.86]	3.85* [2.31, 5.39]	4.40* [2.69, 6.11]	-0.90† [-1.46, -0.34]	-1.72† [-2.43, -1.01]	-2.06† [-2.83, -1.29]	-0.96† [-1.54, -0.39]	-1.22† [-1.83, -0.61]	-0.17† [-0.80, 0.46]	F= 59.69 p<0.001 ηp²= 0.67	F= 196.80 p<0.001 ηp²= 0.69	F= 4.85 p=0.003 ηp²= 0.14
DPP_peak_·BM^−1^ (W·kg^−1^)	2.69† [1.43, 3.95]	1.87† [1.07, 2.67]	2.89† [1.56, 4.20]	2.86† [1.54, 4.18]	-0.80 [-1.35, -0.24]	-1.20* [-1.85, -0.54]	-1.81* [-2.55, -1.08]	-0.55 [-1.10, 0.01]	-1.08* [-1.68, -0.48]	-0.40 [-1.04, 0.23]	F= 28.24 p<0.001 ηp²= 0.49	F= 133.56 p<0.001 ηp²= 0.60	F= 2.00 p=0.119 ηp²= 0.06
VO_2peak_‐DP (mL·min^−1^)	2.61* [1.34, 3.87]	1.62* [0.84, 2.40]	4.28* [2.64, 5.92]	4.38* [2.67, 6.08]	-0.75† [-1.31, -0.19]	-1.52† [-2.21, -0.82]	-1.66† [-2.38, -0.93]	-0.91† [-1.48, -0.34]	-0.98† [-1.57, -0.38]	-0.01† [-0.64, 0.62]	F= 39.03 p<0.001 ηp²= 0.57	F= 169.63 p<0.001 ηp²= 0.66	F= 4.81 p=0.003 ηp²= 0.14
VO_2peak_‐DP·BM^−1^ (mL·kg^−1^ min^−1^)	2.32* [1.11, 3.52]	1.21* [0.47, 1.94]	2.73* [1.44, 4.01]	2.36* [1.15, 3.56]	-0.47† [-1.02, 0.09]	-0.69† [-1.32, -0.06]	-0.97† [-1.64, -0.31]	-0.37† [-0.92, 0.18]	-0.62† [-1.19, -0.04]	-0.16† [-0.79, 0.47]	F= 7.94 p<0.001 ηp²= 0.21	F= 89.90 p<0.001 ηp²= 0.50	F= 3.51 p= 0.019 ηp²= 0.11
DPP_15s_ (W)	2.89† [1.59, 4.19]	2.08† [1.26, 2.91]	2.69† [1.41, 3.97]	2.98† [1.64, 4.32]	-0.92* [-1.48, -0.36]	-2.05* [-2.79, -1.30]	-1.93* [-2.68, -1.18]	-1.07* [-1.65, -0.49]	-0.86* [-1.45, -0.28]	0.22 [-0.41, 0.85]	F= 43.89 p<0.001 ηp²= 0.60	F= 146.24 p<0.001 ηp²= 0.62	F= 1.47 p=0.228 ηp²= 0.05
DPP_15s_ ·BM^−1^ (W·kg^−1^)	3.20† [1.84, 4.57]	2.02† [1.21, 2.84]	1.96† [0.82, 3.11]	2.08† [0.93, 3.23]	-0.89* [-1.45, -0.33]	-1.81* [-2.53, -1.09]	-1.72* [-2.45, -1.00]	-0.81* [-1.38, -0.25]	-0.66 [-1.23, -0.08]	0.18 [-0.45, 0.81]	F= 27.20 p<0.001 ηp²= 0.48	F= 115.19 p<0.001 ηp²= 0.57	F= 0.17 p= 0.92 ηp²= 0.01
Maximal aerobic power and capacity (treadmill running with poles)													
VO_2max_ (mL·min^−1^)	1.95* [0.70, 3.20]	2.02* [1.18, 2.86]	5.40* [3.47, 7.34]	5.72* [3.63, 7.81]	-0.73† [-1.33, -0.14]	-1.57† [-2.30, -0.84]	-1.60† [-2.35, -0.85]	-0.92† [-1.50, -0.35]	-0.96† [-1.56, -0.37]	-0.04† [-0.67, 0.59]	F= 40.70 p<0.001 ηp²= 0.60	F= 195.60 p<0.001 ηp²= 0.70	F= 3.53 p=0.018 ηp²= 0.11
VO_2max_·BM^−1^ (mL·kg^−1^· min^−1^)	1.26† [0.11, 2.40]	1.53† [0.74, 2.32]	2.29† [1.09, 3.50]	2.93† [1.60, 4.27]	-0.40 [-0.99, 0.18]	-0.66 [-1.31, 0.00]	-0.94* [-1.63, -0.25]	-0.31 [-0.86, 0.25]	-0.56 [-1.14, 0.02]	-0.21 [-0.84, 0.42]	F= 5.91 p=0.001 ηp²= 0.18	F= 78.61 p<0.001 ηp²= 0.49	F= 1.46 p= 0.232 ηp²= 0.05

Pairwise comparisons are visualized as heatmap-style tiles, where color reflects the direction of the effect: blue, bias towards males; red, bias towards females; green, bias towards older age groups; yellow, bias towards younger age groups. Green colored tiles reflect significant effects (p < 0.05). * Indicates Bonferroni-adjusted statistically significance *post hoc* differences (p < 0.05). † Indicates that effect sizes are reported for descriptive purposes only as pairwise comparisons were not subjected to *post hoc* analyses based on the corresponding ANOVA results. Abbreviations: 1RM, one‐repetition maximum; BM, body mass; DPP_15s_, mean power during 15s double poling ergometer sprint; DPP_PEAK_, peak power during the double poling ergometry incremental test to exhaustion; VO_2max_, maximal oxygen uptake; VO_2peak_ DP, peak oxygen uptake during double poling ergometry; BM, body mass.

**Figure 2:**
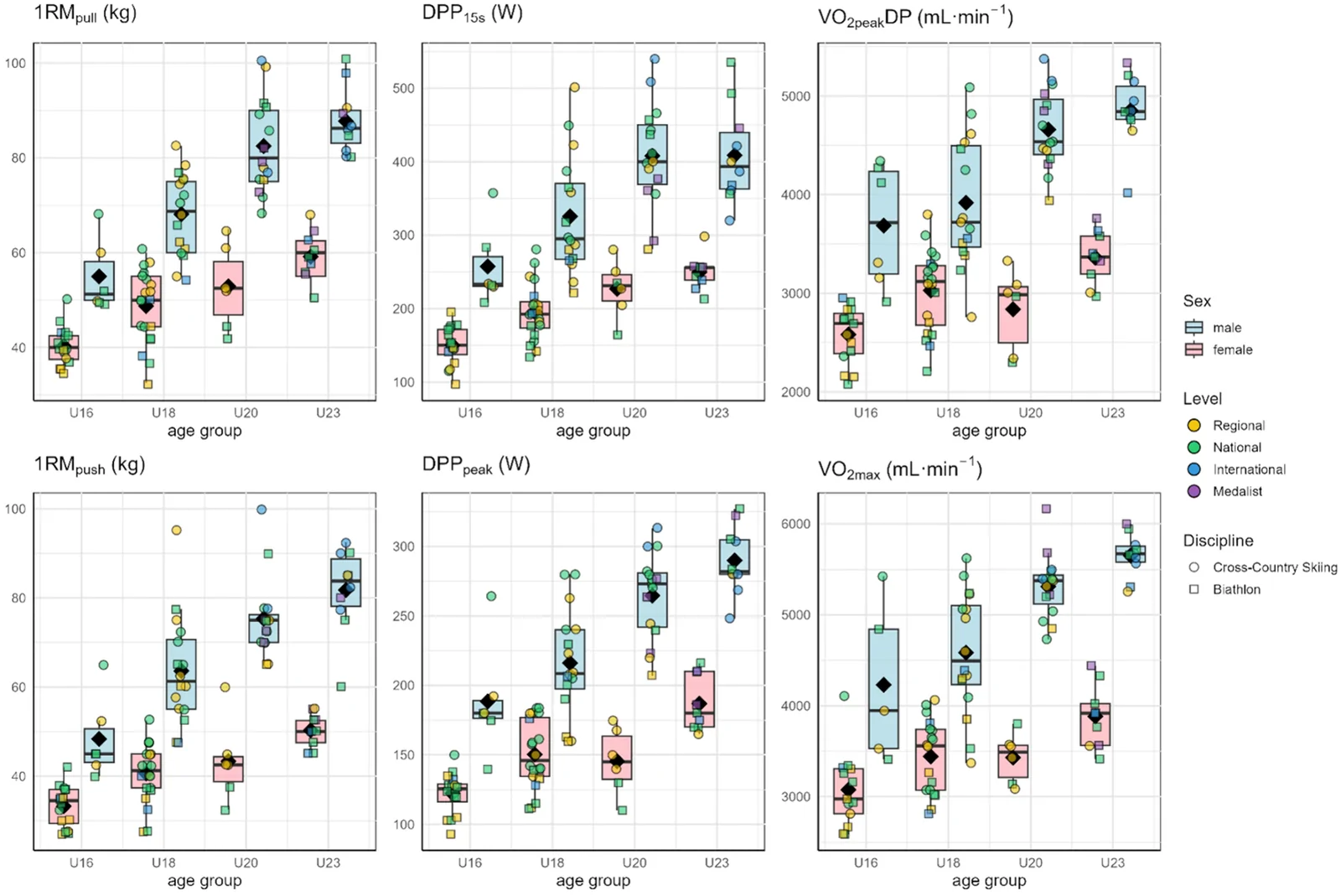
Distribution of maximal strength, power output during DP ergometry, and aerobic power across age groups and sex. Box plots represent the median and interquartile range, with whiskers indicating the data range. Individual data points are overlaid as scatter plots to illustrate inter-individual variability. Diamonds indicate group means. Point color denotes performance level, and point shape indicates sport discipline. Abbreviations: 1RM, one‐repetition maximum; DPP_15s_, mean power during 15s double poling ergometer sprint; DPP_PEAK_, peak power during the double poling ergometry incremental test to exhaustion; VO_2max_, maximal oxygen uptake; VO_2peak_ DP, peak oxygen uptake during double poling ergometry.

For anthropometric characteristics, MANOVA revealed large main effects of age group (F_(6,180)_ = 10.5, p < 0.001, η² _p_= 0.26) and sex (F_(2,89)_ = 88.67, p < 0.001, η²_p_ = 0.67), but no age group × sex interaction (F_(6,180)_ = 2.03, p = 0.064, η²_p_ = 0.06). Follow-up ANOVAs confirmed significant effects of age and sex on height and body mass (all p < 0.001), with males being taller and heavier across all age groups. No significant age group × sex interactions were observed for either measure. *Post-hoc* comparisons showed that body mass differed between all age groups except U20 and U23, whereas height differences were primarily observed between the two younger and the two older age groups.

Upper-body maximal strength, assessed via 1RM in bench press and bench pull, showed large main effects of age group (MANOVA_PILLAI_; F_(12,267)_ = 8.03, p < 0.001, η²_p_ = 0.27) and sex (F_(4,87)_ = 56.95, p < 0.001, η²_p_ = 0.72), whereas the age group × sex interaction was not significant (F_(12,267)_ = 1.47, p = 0.137, η²_p_ = 0.06). Absolute 1RM_press_ and 1RM_pull_ were higher in males and increased with age, with significant univariate age × sex interactions for both measures (p = 0.01, η²_p_ = 0.13 and 0.12, respectively). The interaction reflected variation in the magnitude of sex differences across age groups, with the largest differences observed in U20 and U23. In contrast, ANOVA revealed no interaction for body mass normalized strength measures (p ≥ 0.404). *Post-hoc* comparisons for normalized strength measures revealed significant age-related differences between all age groups, except for the U20 vs U23 comparison.

For upper-body endurance performance, MANOVA_PILLAI_ revealed large main effects of age group (F(_12,267)_ = 9.08, p < 0.001, η²_p_ = 0.29), and sex (F_(6,87)_ = 55.49, p < 0.001, η²_p_ = 0.72), as well as a significant age group × sex interaction (F_(12,267)_ = 2.07, p = 0.019, η²_p_ = 0.09). Males showed higher performance across age groups, with significant univariate age × sex interactions for absolute DPP_peak_ (p = 0.003, η²_p_ = 0.14), VO_2peak_ DP (p = 0.003, η²_p_ = 0.14), and VO_2peak_ DP·BM^−1^ (p = 0.019, η²_p_ = 0.11), indicating variation in the magnitude of sex differences across age groups, with larger differences observed in the older age groups. In contrast, no significant interaction was observed for DPP_15s_ and DPP_15s_·BM^−1^ (p = 0.228 and 0.920, respectively).

Maximal aerobic power showed large main effects of age group (MANOVA_PILLAI_; F(_12,240)_ = 5.91, p < 0.001, η²_p_ = 0.23) and sex (F_(4,87)_ = 50.28, p < 0.001, η²_p_ = 0.72), together with a significant age group × sex interaction (F_(12,240)_ = 2.32, p = 0.008 η²_p_ = 0.10). VO_2max_ increased with age and was higher in males across age groups. Follow-up ANOVAs revealed a significant interaction for VO_2max_ (p = 0.018, η²_p_ = 0.11), but not for VO_2max_·BM^−1^ (p = 0.232), indicating that the variation in sex differences across age groups was attenuated after normalization to body mass.

For non-significant pairwise comparisons, TOST was conducted using predefined equivalence bound of ± 0.50 SD. None of the comparisons met the predefined criterion for practical equivalence (TOST p-values: 0.15 to 0.86), indicating that non-significant findings could not be interpreted as evidence of practical equivalence.

Fractional utilization of VO_2max_ during DP, expressed both as the absolute difference between VO_2max_ and VO_2peak_ DP (Δ) and as ratio of VO_2peak_ DP·VO_2max_^-1^, is presented by sex within each age group: In U16 athletes, the VO_2peak_ DP·VO_2max_^-1^ ratio was 0.9 ± 0.13 (Δ = 661 ± 325 mL·min^-1^) in males and 0.85 ± 0.08 (Δ = 423 ± 119 mL·min^-1^) in females. Corresponding values in U18 were 0.86 ± 0.06 (Δ = 623 ± 60 mL·min^-1^) and 0.89 ± 0.08 (Δ = 394 ± 248 mL·min^-1^), respectively. In U20 males exhibited a ratio of 0.88 ± 0.06 (Δ = 652 ± 321 mL·min^-1^), whereas females showed a ratio of 0.83 ± 0.03 (Δ = 592 ± 250 mL·min^-1^). For U23 athletes, the ratio was 0.86 ± 0.04 (Δ = 800 ± 209 mL·min^-1^) in males and 0.87 ± 0.05 (Δ = 522 ± 225 mL·min^-1^) in females (**Figure 2**). MANOVA_PILLAI_ revealed a large effect of sex (η²_p_= 0.43) and moderate effects of age (η²_p_= 0.12), along with a significant age-by-sex interaction (η²_p_= 0.08), indicating that sex differences increased with age. Follow-up ANOVAs demonstrated significant effects of sex (η²_p_= 0.15) and age (η²_p_= 0.10) for the absolute difference between VO_2max_ and VO_2peak_ DP, whereas no significant main or interaction effects were observed for relative utilization, suggesting a stable proportion of aerobic capacity used during DP across the sample.

Statistical assumptions were assessed using Shapiro-Wilk, Levene’s, and Box’s M tests, with appropriate robust procedures applied where assumptions were violated. Detailed results are provided in the **Supplementary Materials**.

### 3.2 Correlation analysis

Partial correlation analyses demonstrated moderate to strong associations between upper-body maximal strength and DP performance after adjustment for age and sex. Scatter plots and partial Pearson correlation coefficients for absolute values are illustrated in **Figure 3**, while body mass normalized associations are presented in **Supplementary Materials**.

**Figure 3:**
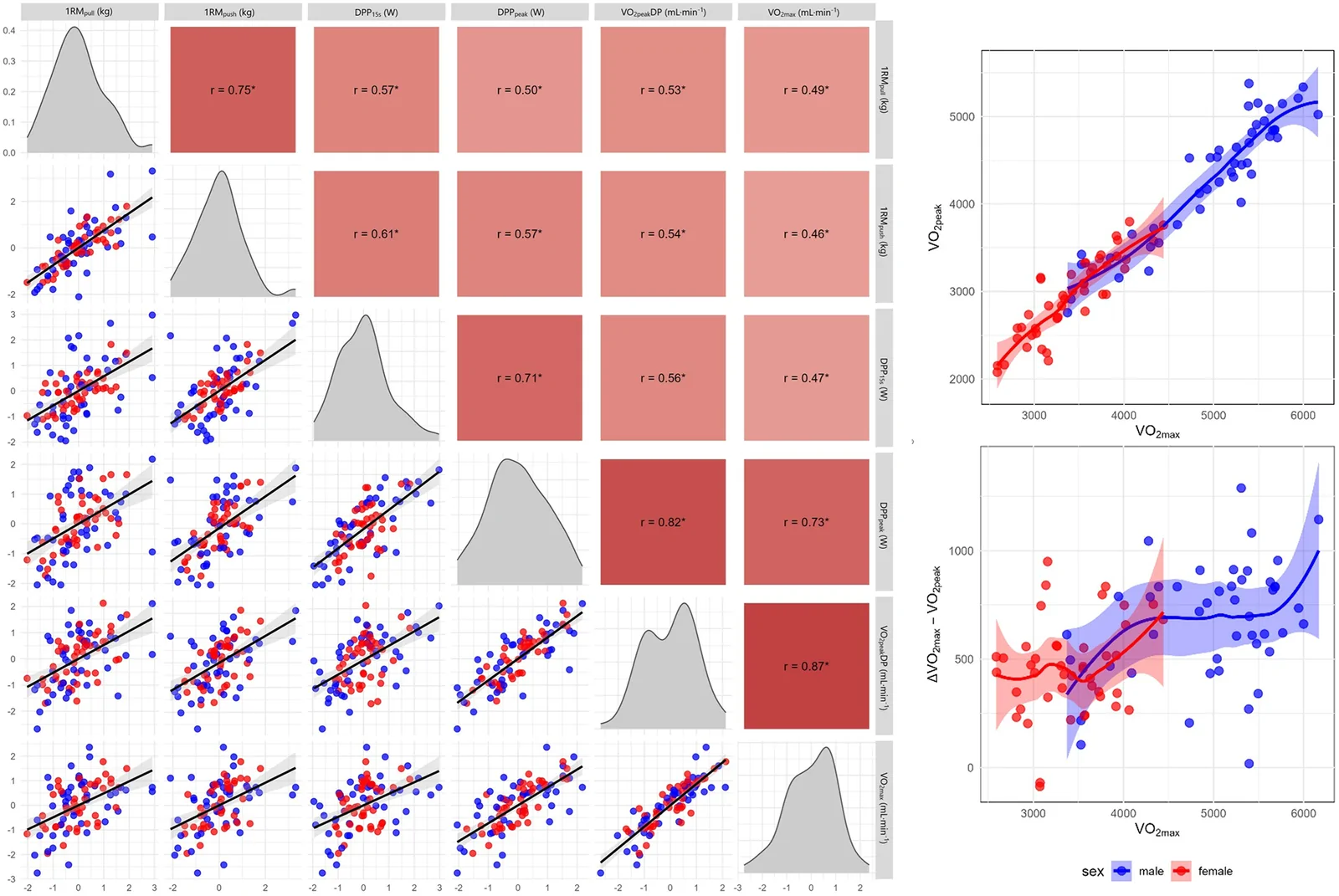
Combined visualization of relationship between maximal strength, DP performance, and aerobic power. The lower triangle of the pairwise scatterplot displays residualized and z-standardized scatterplots of sex- and age-adjusted residuals, with linear regression lines (black) and 95% confidence intervals (grey). Diagonal panels depict kernel density estimates of standardized residuals. The upper triangle presents partial Pearson correlation coefficients (r), controlling for sex and age, visualized as heatmap-style tiles (blue = negative, red = positive). Additionally separate scatterplots visualize fractional utilization of VO2max during DP with points colored by sex (male = blue, female = red), along with LOESS trend lines and colored 95% confidence intervals computed per sex. Abbreviations: 1RM, one‐repetition maximum; DPP_15s_, mean power during 15s double poling ergometer sprint; DPP_PEAK_, peak power during the double poling ergometry incremental test to exhaustion; VO_2max_, maximal oxygen uptake; VO_2peak_ DP, peak oxygen uptake during double poling ergometry.

Absolute 1RM bench press and bench pull were significantly associated with DPP_15s_ (r = 0.58 – 0.60, p < 0.05), DPP_peak_ (r = 0.51 – 0.59, p < 0.05), and VO_2peak_ DP (r = 0.52 – 0.53, p < 0.05). After normalization to body mass, associations between 1RM and DPP_15s_ (r = 0.27 – 0.38, p < 0.05) and DPP_peak_ (r = 0.25 – 0.39, p < 0.05) decreased. Associations with VO_2peak_ DP were also attenuated after normalization (r = 0.24, p < 0.05 and r = 0.13, p > 0.05, respectively).

Aerobic power showed stronger associations with DPP_peak_ (r = 0.73 – 0.80, p < 0.05) than with DPP15s (r = 0.51 – 0.61, p < 0.05) for absolute measures. After normalization to body mass, the associations with DPP_peak_ remained moderate to strong (r = 0.52 – 0.65, p < 0.05), whereas associations with DPP_15s_ were weak and non-significant (r = 0.08 – 0.17, p > 0.05). Overall, the correlations analysis indicated stronger associations of maximal strength with DP_15s_, while aerobic power was more strongly associated with DPP_peak_.

Notably, unadjusted correlations were consistently stronger than the corresponding partial correlations. Across absolute measures, associations of 1RM with DPP_15s_, DPP_peak_, and VO_2peak_ DP ranged from r = 0.88 – 0.91, (p > 0.05). The attenuation after adjustments suggests that part of the observed associations may reflect age-and sex-related differences, which are also apparent in scatter plots. Complete unadjusted correlations coefficients and corresponding scatterplots are provided in the **Supplementary Materials**.

### 3.3 Preliminary analysis

Preliminary MANOVAs revealed statistically significant multivariate effects of sport discipline and performance level. However, follow-up univariate analyses showed only small to moderate effects (η²_p_= 0.00 – 0.16), and scatterplots indicated substantial overlap between groups. Chi^2^ analyses confirmed that age and sex were evenly distributed across discipline and performance levels (χ^2^ = 0.05 – 7.68, p= 0.05 – 0.94; Cramer’s V = 0.01 – 0.29). Based on these findings, data was pooled across disciplines and performance levels for the main analyses.

## 4 Discussion

The present study characterized age- and sex-related differences in upper-body strength, aerobic power, and double poling ergometer performance among adolescent and junior cross-country skiers and biathletes and examined the associations between DP performance and upper-body strength independent of age and sex. The analyses revealed large main effects of both age and sex on all upper-body performance measures. Older athletes consistently outperformed younger athletes, while sex differences in upper-body performance increased with age in absolute terms. Through normalization to body mass, however, sex differences remained of similar magnitude across age groups.

### 4.1 Age- and sex-related differences

The pronounced sex differences in upper-body strength and DP performance found in the current study have also been consistently reported in previous studies, but the magnitude of these differences appears to depend on age, exercise mode, and how performance is expressed. In a previous study sex differences of 19% to 36% in upper-body maximal strength, 31% to 33% in DP power, and 29% in VO2peak DP were reported (Wagner et al., 2025). The population comprised cross-country skiers and biathletes aged 16.7 ± 1.7 years. The magnitude of these sex differences was most comparable to that observed in the younger age groups of the present study. In contrast, Sollie and Losnegard (2022) observed relatively stable sex differences across adolescent, junior, and senior cross-country skiers. However, their study examined G2 roller-ski skating and reported values relative to body mass, whereas the current study examined upper-body dominant DP and reported performance in both absolute and body mass normalized terms. In adult elite skiers, Hegge, Bucher (Hegge et al., 2016) reported even more pronounced sex differences in power production (87%) and VO_2peak_ (51%) during DP ergometry in adult elite skiers. Taken together, the present study extends previous research by demonstrating how sex differences in upper-body performance vary across adolescence and early adulthood within a single athletic population, while also showing that these age-related differences are largely attenuated when performance is normalized to body mass.

The differences between the present findings and those of Sollie and Losnegard (2022) may have several explanations. First, DP relies more strongly on upper-body force production than G2 skating, which may make sex differences in upper-body muscle mass and strength more apparent (Kuipers et al., 1985; Hegge et al., 2016; Skattebo et al., 2025). Second, expressing performance in absolute or body-mass-normalized terms can substantially influence the magnitude of the observed sex differences. This is also reflected in the present results, where most age × sex interactions were evident for absolute but not body-mass-normalized measures. Differences in performance level, training background, and sample composition may also have contributed to the contrasting findings. Since absolute power output and VO_2peak_ DP are strongly related to fat-free mass (Alsobrook and Heil, 2009; Cohen, 2013; Wagner et al., 2025), the sex-specific physiological changes during puberty may partly explain the larger absolute performance differences observed in the older age groups. In males, rising testosterone concentrations during puberty promote greater gains in muscle mass and lower body fat, whereas comparable changes in body composition are less pronounced in females. The divergent changes in body size and muscle mass between sexes across adolescence may therefore contribute to a widening of the performance gap between sexes, even at relative performance levels (Joyner, 2017; Sandbakk et al., 2018). Although a significant age × sex interaction was detected for body mass normalized VO_2peak_ DP, this effect was likely driven by unusually lower values in the female U20 group and should be interpreted with caution given the small sample size (n= 6) and performance level distribution within this subgroup. More generally, because of the cross-sectional design, differences between age groups should not be interpreted as individual developmental changes over time.

In the present study, older athletes consistently outperformed younger age groups. These age-related improvements in upper-body performance were evident across all performance measures and reflect a combination of biological maturation, accumulated training exposure, and increased neuromuscular performance (Joyner, 2017; Hunter et al., 2023). The structured training environments of the participants, including regular upper-body endurance and resistance training, likely contributed to progressive improvements in strength and aerobic power with advancing age (Lloyd et al., 2015a; Lloyd et al., 2015b). However, performance-based selection processes in competitive sport may also influence observed age-group differences by disproportionately retaining higher-performing athletes in older categories.

### 4.2 Upper-body strength and DP performance

A central finding of this study is the strong association between absolute upper-body maximal strength and DP ergometer performance. Both 1RM bench press and bench pull were significantly correlated with DPP_15s_, DPP_peak_ and VO_2peak_ DP, even after adjusting for age and sex. These findings are consistent with previously reported associations between upper-body maximal strength and DP performance (r = 0.52 – 0.89; sex-adjusted: r = 0.50 – 0.82) (Osteras et al., 2016; Sunde et al., 2019; Wagner et al., 2025). In contrast, when strength measures were normalized to body mass, the strength-performance associations were substantially reduced and, in case of VO_2peak_ DP, no longer statistically significant.

The stronger association observed for absolute strength may reflect the high force impulsesrequired to achieve high skiing velocities within a limited time for force application (Stöggl et al., 2011). However, this should not be interpreted as evidence that relative strength is of limited relevance to on-snow skiing, where performance also depends on propelling the athlete’s body mass across varying terrain. The attenuation of the strength-performance associations following normalization to body mass may partly reflect the removal of performance-relevant variance related to morphological characteristics (i.e., muscle mass), rather than a lack of physiological relevance of relative strength. In addition, differences in participant characteristics, sample size, and the protocols used to assess strength and DP performance may contribute to the heterogeneous associations reported in the literature. For example, Stöggl et al. (2011) reported non-significant relationships between absolute 1RM strength and peak DP velocity (r= 0.24 – 0.51). Such differences may partly reflect the performance outcomes assessed, as skiing velocity depends not only on force generation capacity but also on the timing and technical application of force (Stoggl and Muller, 2009; Stöggl et al., 2011). Thus, within trained and relatively homogenous populations, more ski-specific approaches may be needed to capture the relevance of maximal strength in the context of coordinated, sport-specific force application. Overall, maximal strength should not be considered an isolated determinant of DP performance, but rather as one component interacting with muscle mass, aerobic power and skiing technique (Stöggl et al., 2011; Osteras et al., 2016).

### 4.3 Aerobic power and fractional utilization of VO_2max_

Aerobic power also emerged as a significant predictor of DP performance, particularly for peak power output during the incremental test. However, its association with sprint performance was weaker, becoming non-significant after normalization for body mass, indicating that short-duration, high-intensity efforts are more dependent on maximal strength than aerobic capacity. These findings align with previous research, where neuromuscular capacity emerged as key contributor to sprint performance (Douglas et al., 2021; Martin-Rodriguez et al., 2024; Keiner et al., 2025). Notably, increases in absolute aerobic power during adolescence have been shown to be associated with increases in fat-free mass, and thereby strength (Armstrong and Welsman, 2019; Landgraff et al., 2021). Thus, muscle mass and strength not only determine short-duration, high-intensity exercise performance but also indirectly shapes aerobic power development during adolescents, thereby influencing the capacity for sustained (upper-body) endurance performance.

Consistent with previous research, the fractional utilization of VO_2max_ during DP (VO_2peak_ DP · VO_2max_^-1^) did not differ significantly across age groups or between sexes (Losnegard, 2019; Wagner et al., 2025). This suggests that, despite large differences in absolute VO_2peak_ values, the proportion of maximal aerobic capacity utilized during DP remains relatively stable. This finding may reflect the greater contribution of peripheral limitations to upper-body aerobic performance compared with lower body exercise. In cross-country skiers, oxygen extraction has been shown to be lower in the arm than in the legs during exercise, potentially due to differences in blood flow distribution and muscle oxygen diffusion, while aerobic performance is also related to the amount of active muscle mass. These peripheral differences may therefore contribute to the lower VO_2peak_ DP compared with lower-body exercise, where central cardiovascular function limits performance (Astrand and Saltin, 1961; Shephard et al., 1988; di Prampero, 2003; Calbet et al., 2005; Larsen et al., 2016). However, despite this overall agreement, substantial inter-individual variability was evident (see **Figure 3**), with no discernible systematic pattern. Some evidence suggests that VO_2peak_ DP and the fractional utilization of VO_2max_ during DP can be enhanced through upper-body-specific interventions (Terzis et al., 2006; Wagner et al., 2024). However, the underlying mechanisms of this phenomenon remain incompletely understood and further research is required to elucidate the physiological mechanisms underpinning these adaptations. It should also be noted that fractional utilization ratios do not capture absolute differences between VO_2peak_ DP and VO_2max_. Identical ratios at higher absolute VO_2_ values imply a greater absolute capacity for performance improvement (see **Figure 3**), suggesting that athletes with superior aerobic profiles may benefit more substantially from targeted upper-body training interventions (Terzis et al., 2006).

### 4.4 Unexpected and null findings

Notably, no statistically significant age-related differences were observed between the U20 and U23 age groups. This finding may indicate that differences in upper-body performance begin to plateau from late adolescence into early adulthood, as the major sex-specific physiological changes associated with puberty and post-puberty are largely established by this stage. Furthermore, as athletes approach adulthood, a greater proportion of their sport-specific performance potential may already have been developed, potentially resulting in smaller subsequent improvements in performance. However, the non-significant equivalence tests do not support practical equivalence between U20 and U23 athletes. The overlap in training and performance levels between these groups, together with the limited group-specific sample sizes, may also have contributed to the absence of significant differences. Another unexpected finding was the absence of an age-by-sex interaction for DP sprint performance. Given the known influence of maximal strength on short-duration efforts, a greater divergence with age might have been anticipated. However, DPP15s depends not only on maximal strength but also on the rapid and coordinated application of force within the DP movement (Stoggl and Muller, 2009; Stöggl et al., 2011). The body-mass-scaled drag factor may also have influenced sprint performance, although this alone is unlikely to explain the finding because age × sex interactions were observed for other DP-related outcomes. A further notable finding was the absence of age or sex differences in the relative utilization of VO_2max_ during DP. This may indicate that VO_2peak_ DP and VO_2max_ vary broadly in parallel across age and sex, consistent with previous observations in cross-country skiers (Losnegard, 2019; Hansen et al., 2021; Wagner et al., 2025). However, the ratio may mask differences in absolute aerobic capacity, as the absolute difference between VO_2max_ and VO_2peak_ DP showed significant age and sex effects in the present study.

### 4.5 Strength and limitations

This study has several strengths, including a relatively large and well-balanced overall sample, comprehensive and standardized testing protocols, and robust statistical analyses, providing a broader basis for the observed associations compared with previous studies using smaller samples. However, some limitations should be acknowledged. The cross-sectional design precludes causal inferences about developmental or training-related adaptations. Biological maturation was not directly assessed, which may have introduced variability in the interpretation of age-related effects. Therefore, differences between age groups may partly reflect variation in maturational status rather than chronological age alone. Moreover, maximal strength and performance were normalized to total body mass. While this is a practical approach, it does not distinguish between changes in fat-free mass and fat mass that occur with biological maturation. Alternative approaches, such as normalization to fat-free mass, may provide more accurate estimates of sex- and age-related differences in strength and performance and may help to better understand the extent to which observed sex differences are related to differences in muscle mass and body composition. Despite the relatively large overall sample, some age x sex subgroups were small, particularly the female U20 group (n = 6). Estimates within these subgroups should therefore be interpreted with caution, as individual observations and differences in performance level may have had a greater influence on the observed effects. Furthermore, cross-country skiers and biathletes were pooled for the main analysis. Although preliminary analyses showed substantial overlap between disciplines and no relevant imbalance in age and sex distribution, potential discipline-specific differences in training background and physiological characteristics cannot be completely excluded. Additionally, while the use of a DP ergometer provides a controlled and reliable measure of upper-body performance, it may not fully capture the technical, environmental, and psychological demands of on-snow skiing. Moreover, the body-mass-scaled drag factor used during DP ergometry may have influenced the mechanical demands across athletes and should particularly be considered when interpreting the DPP15s results. Nonetheless, the controlled testing environment can be considered a methodological strength, as it minimizes external confounding factors. Considering the limited availability of longitudinal data in youth endurance athletes, future studies should follow athletes across age categories and include assessments of biological maturation to better distinguish age-related differences from training adaptations and selection processes. Despite these limitations, the present findings provide meaningful insight into the complex relationships between maximal upper-body strength, aerobic power, and DP ergometer performance across age groups and sexes.

### 4.6 Practical applications

From a practical perspective, the present findings support the use of upper-body maximal strength as one component of performance monitoring in adolescent and junior Nordic Skiers. However, the stronger associations observed for absolute compared with body-mass normalized strength should not be interpreted as indicating that relative strength or power measures are unnecessary. Particularly during adolescence, when substantial changes in body mass and body composition occur, monitoring absolute and relative performance measures alongside body mass and body composition may provide a more informed basis for training decisions. Notably, the present study does not establish the extent to which changes in maximal strength or body compositions are associated with meaningful changes in on-snow performance. Therefore, although resistance training in adolescence is generally considered safe when appropriately designed and supervised (Faigenbaum et al., 2009; Lloyd et al., 2014), these findings should not be used to prescribe a specific resistance training approach or to assume that increasing maximal strength will directly improve DP performance or reduce sex differences. Given the cross-sectional design, the practical implications should be considered as potential considerations for training rather than evidence for specific training effects.

## 5 Conclusion

The present study identified age- and sex-related differences in upper-body strength and DP performance that appeared more pronounced than previously reported for lower-body performance. Older athletes consistently outperformed younger athletes, while sex differences in upper-body performance were larger in the older age groups for several absolute measures. However, following adjustment for body mass, most age x sex interactions were no longer significant. Absolute upper-body strength was consistently associated with DP performance after accounting for age and sex, suggesting that it represents an important performance-related factor in adolescent and junior Nordic skiers. However, given the cross-sectional design of the study, it is not possible to make causal inferences regarding training adaptations or physiological development. Longitudinal and intervention studies are needed to determine whether changes in upper-body strength translate into improved DP performance in male and female athletes.

## Data Availability

The raw data supporting the conclusions of this article will be made available by the authors, without undue reservation.
