# Supplementary material for: Age-related widening of sex differences in upper-body strength and double poling performance in U16-U23 cross-country skiers and biathletes
Source: Front Physiol. 2026 Oct 27;17:1890471. doi: 10.3389/fphys.2026.1890471 (PMC13747838; doi:10.3389/fphys.2026.1890471)
Supplement: Supplementary file 1 [file DataSheet1.docx]

Supplementary Material

# 1 Supplementary Data

## 1.1 Preliminary, Analysis

Preliminary MANOVAs revealed significant multivariate effects of discipline (F_(14,74)_= 2.51, p=0.005, η²ₚ= 0.32) and performance level (F_(14,74)_= 2.62, p= 0.004, η²ₚ= 0.33).

However, follow-up univariate ANOVAs showed only small to moderate effects across individual variables (discipline, η²ₚ= 0.00 – 0.08; performance level, η²ₚ= 0.01 – 0.16), suggesting that the multivariate significance was predominantly driven by small, dispersed differences rather than clearly distinct performance profiles. Scatterplots further confirmed substantial overlap between groups.

| **Variable** | **ANOVA (discipline)** | | |
| --- | --- | --- | --- |
|  | **F** | **p** | **η²ₚ** |
| **Size  (m)** | 3.08 | 0.08 | 0.03 |
| **Weight  (kg)** | 7.94 | 0.01 | 0.08 |
| **1RM bench pull  (kg)** | 6.09 | 0.02 | 0.07 |
| **1RM·BM^−1^ bench pull (kg·kg−1)** | 3.49 | 0.07 | 0.04 |
| **1RM bench press  (kg)** | 7.60 | 0.01 | 0.08 |
| **1RM·BM^−1^ bench press (kg·kg^−1^)** | 5.55 | 0.02 | 0.06 |
| **DPP_peak_  (W)** | 7.03 | 0.01 | 0.07 |
| **DPP_peak_·BM^−1^  (W·kg^−1^)** | 5.59 | 0.02 | 0.06 |
| **VO_2peak_‐DP  (mL·min^−1^)** | 6.99 | 0.01 | 0.07 |
| **VO_2peak_‐DP·BM^−1^ (mL·kg−1 min^−1^)** | 4.64 | 0.03 | 0.05 |
| **DPP_15s_  (W)** | 6.58 | 0.01 | 0.07 |
| **DPP_15s_ ·BM^−1^  (W·kg^−1^)** | 4.65 | 0.03 | 0.05 |
| **VO_2max_  (mL·min^−1^)** | 3.24 | 0.08 | 0.04 |
| **VO_2max_·BM^−1^  (mL·kg^−1^· min^−1^)** | 0.20 | 0.65 | 0.00 |

| **Variable** | **ANOVA (performance level)** | | |
| --- | --- | --- | --- |
|  | **F** | **p** | **η²ₚ** |
| **Size  (m)** | 0.87 | 0.35 | 0.01 |
| **Weight  (kg)** | 0.58 | 0.45 | 0.01 |
| **1RM bench pull  (kg)** | 1.33 | 0.25 | 0.02 |
| **1RM·BM^−1^ bench pull (kg·kg−1)** | 1.51 | 0.22 | 0.02 |
| **1RM bench press  (kg)** | 2.05 | 0.16 | 0.02 |
| **1RM·BM^−1^ bench press (kg·kg^−1^)** | 2.55 | 0.11 | 0.03 |
| **DPP_peak_  (W)** | 6.55 | 0.01 | 0.07 |
| **DPP_peak_·BM^−1^  (W·kg^−1^)** | 11.53 | <0.01 | 0.12 |
| **VO_2peak_‐DP  (mL·min^−1^)** | 5.57 | 0.02 | 0.06 |
| **VO_2peak_‐DP·BM^−1^ (mL·kg−1 min^−1^)** | 16.00 | <0.01 | 0.06 |
| **DPP_15s_  (W)** | 2.20 | 0.14 | 0.02 |
| **DPP_15s_ ·BM^−1^  (W·kg^−1^)** | 2.81 | 0.10 | 0.03 |
| **VO_2max_  (mL·min^−1^)** | 5.23 | 0.02 | 0.06 |
| **VO_2max_·BM^−1^  (mL·kg^−1^· min^−1^)** | 10.23 | <0.01 | 0.11 |

## 1.2 Main Analysis, statistical details

### 1.2.1 MANOVA

The Shapiro-Wilk test indicated normality (p > 0.05) for all dependent variable within each group, except for 1RM_pull_ in U16 males (p= 0.048) and both 1RM_press_ (p= 0.006) and VO_2max_·BM^-1^ (p= 0.049) in U20 males. However, visual inspection of distributions did not reveal systematic deviations from normality. Therefore, the assumption of multivariate normality was considered to be sufficiently met for the MANOVAs. Box’s M test indicated violations of the homogeneity of covariance matrices for the domains of maximal upper-body strength, upper-body endurance, and maximal aerobic power (all p<0.001). Consequently, Pillai’s trace was used as a robust multivariate test statistic.

### 1.2.2 ANOVA

Levene’s test and F_max_ statistics indicated that the assumption of homogeneity of variances was met for all dependent variables except for 1RM_pull_ (p= 0.048, F_max_ = 6.4), DPP_15s_ (p= 0.020; F_max_= 11.5), and VO_2max_ (p= 0.002; F_max_= 13.4). For these variables, additional Welch’s ANOVA and Welch’s t-tests were conducted as robustness checks. As the robustness analyses yielded a similar significance pattern as the standard two-way ANOVAs, the latter was reported as the primary results.

### 1.2.3 Correlation Analyses

The Shapiro-Wilk test showed significant deviations from normality for all absolute variables in the overall sample (p < 0.01). However, the residualized variables, after controlling for age and sex, did not significantly deviate from normality (all p ≥ 0.067). After normalization for body mass no deviations from normality were detected, both in the overall sample and after residualization (p>0.05).

# 2 Supplementary Figures and Tables

## 2.1 Discipline-specific descriptive data

| **Variable** | **Age Group** | **Cross-Country Skiing** | | **Biathlon** | |
| --- | --- | --- | --- | --- | --- |
|  |  | **Female** | **Male** | **Female** | **Male** |
| **Size (m)** | U16 | 1.65 ± 0.11 | 1.74 ± 0.08 | 1.63 ± 0.05 | 1.81 ± 0.06 |
|  | U18 | 1.68 ± 0.05 | 1.79 ± 0.06 | 1.63 ± 0.06 | 1.79 ± 0.03 |
|  | U20 | 1.66 ± 0.04 | 1.86 ± 0.04 | 1.65 ± 0.04 | 1.83 ± 0.06 |
|  | U23 | 1.74 | 1.82 ± 0.03 | 1.70 ± 0.03 | 1.83 ± 0.04 |
| **Weight (kg)** | U16 | 53.5 ± 13.3 | 60.3 ± 9.6 | 52.8 ± 5.6 | 59.5 ± 6.1 |
|  | U18 | 60.6 ± 5.4 | 67.2 ± 8.3 | 52.0 ± 5.1 | 62.9 ± 5.7 |
|  | U20 | 62.3 ± 4.4 | 76.7 ± 4.5 | 56.9 ± 10.0 | 71.6 ± 4.1 |
|  | U23 | 67.7 | 73.4 ± 2.9 | 61.7 ± 4.4 | 75.7 ± 5.6 |
| **1RM bench pull**  **(kg)** | U16 | 42.5 ± 6.6 | 59.2 ± 8.8 | 39.8 ± 3.3 | 50.8 ± 1.4 |
|  | U18 | 53.1 ± 4.7 | 71.0 ± 8.3 | 42.2 ± 6.2 | 63.3 ± 7.7 |
|  | U20 | 57.5 ± 6.1 | 82.8 ± 11.8 | 43.8 ± 1.8 | 82.1 ± 8.0 |
|  | U23 | 67.5 | 85.0 ± 4.0 | 58.1 ± 4.8 | 90.5 ± 8.4 |
| **1RM·BM^−1^ bench pull**  **(kg·kg^−1^)** | U16 | 0.81 ± 0.07 | 0.99 ± 0.10 | 0.76 ± 0.07 | 0.86 ± 0.08 |
|  | U18 | 0.88 ± 0.07 | 1.06 ± 0.10 | 0.81 ± 0.12 | 1.01 ± 0.06 |
|  | U20 | 0.92 ± 0.04 | 1.08 ± 0.15 | 0.78 ± 0.11 | 1.15 ± 0.08 |
|  | U23 | 1.00 | 1.16 ± 0.05 | 0.95 ± 0.11 | 1.20 ± 0.17 |
| **1RM bench press**  **(kg)** | U16 | 32.5 ± 5.0 | 53.3 ± 11.3 | 33.4 ± 4.7 | 43.3 ± 2.9 |
|  | U18 | 44.4 ± 4.1 | 66.8 ± 12.2 | 34.9 ± 5.5 | 58.3 ± 11.7 |
|  | U20 | 47.5 ± 8.4 | 75.8 ± 9.9 | 35.0 ± 3.5 | 74.6 ± 8.4 |
|  | U23 | 55.0 | 85.5 ± 6.0 | 49.7 ± 3.6 | 78.0 ± 11.5 |
| **1RM·BM^−1^ bench press**  **(kg·kg^−1^)** | U16 | 0.62 ± 0.08 | 0.88 ± 0.10 | 0.63 ± 0.08 | 0.74 ± 0.12 |
|  | U18 | 0.74 ± 0.07 | 1.00 ± 0.14 | 0.68 ± 0.12 | 0.92 ± 0.12 |
|  | U20 | 0.77 ± 0.12 | 0.99 ± 0.13 | 0.63 ± 0.17 | 1.04 ± 0.09 |
|  | U23 | 0.81 | 1.17 ± 0.11 | 0.81 ± 0.08 | 1.04 ± 0.18 |
| **DPP_15s_**  **(W)** | U16 | 125.7 ± 16.8 | 273.7 ± 72.2 | 155.5 ± 25.5 | 241.0 ± 38.3 |
|  | U18 | 211.4 ± 37.6 | 349.1 ± 88.5 | 171.6 ± 29.4 | 286.2 ± 49.6 |
|  | U20 | 240.5 ± 32.1 | 434.8 ± 60.2 | 199.5 ± 50.2 | 367.5 ± 72.3 |
|  | U23 | 298.0 | 377.8 ± 38.9 | 243.5 ± 16.4 | 439.6 ± 77.6 |
| **DPP_15s_ ·BM^−1^**  **(W·kg^−1^)** | U16 | 2.4 ± 0.3 | 4.5 ± 0.4 | 2.9 ± 0.4 | 4.0 ± 0.5 |
|  | U18 | 3.5 ± 0.6 | 5.2 ± 1.0 | 3.3 ± 0.5 | 4.6 ± 0.7 |
|  | U20 | 3.9 ± 0.5 | 5.7 ± 0.7 | 3.6 ± 1.5 | 5.1 ± 0.9 |
|  | U23 | 4.4 | 5.2 ± 0.6 | 4.0 ± 0.4 | 5.8 ± 1.0 |
| **DPP_peak_**  **(W)** | U16 | 133.7 ± 14.4 | 212.0 ± 45.4 | 119.7 ± 14.2 | 165.0 ± 21.8 |
|  | U18 | 162.8 ± 18.4 | 230.8 ± 37.7 | 131.8 ± 21.4 | 191.7 ± 26.8 |
|  | U20 | 158.3 ± 16.1 | 276.1 ± 29.1 | 120.0 ± 14.1 | 247.3 ± 28.6 |
|  | U23 | 165.0 | 276.2 ± 20.3 | 189.6 ± 19.3 | 303.6 ± 21.4 |
| **DPP_peak_·BM^−1^**  **(W·kg^−1^)** | U16 | 2.5 ± 0.3 | 3.5 ± 0.2 | 2.3 ± 0.2 | 2.8 ± 0.1 |
|  | U18 | 2.7 ± 0.3 | 3.4 ± 0.3 | 2.5 ± 0.3 | 3.1 ± 0.4 |
|  | U20 | 2.6 ± 0.3 | 3.6 ± 0.4 | 2.1 ± 0.1 | 3.5 ± 0.4 |
|  | U23 | 2.4 | 3.8 ± 0.3 | 3.1 ± 0.2 | 4.0 ± 0.3 |
| **VO_2peak_‐DP**  **(mL·min^−1^)** | U16 | 2471 ± 155 | 3603 ± 644 | 2598 ± 305 | 3769 ± 745 |
|  | U18 | 3266 ± 261 | 4311 ± 725 | 2671 ± 338 | 3595 ± 440 |
|  | U20 | 2940 ± 424 | 4722 ± 402 | 2633 ± 473 | 4566 ± 425 |
|  | U23 | 3007 | 4873 ± 189 | 3404 ± 254 | 4832 ± 516 |
| **VO_2peak_‐DP·BM^−1^**  **(mL·kg^−1^ min^−1^)** | U16 | 54.0 ± 4.9 | 59.6 ± 1.4 | 48.4 ± 5.0 | 62.9 ± 6.4 |
|  | U18 | 54.1 ± 4.9 | 61.4 ± 7.2 | 51.2 ± 2.7 | 56.6 ± 3.9 |
|  | U20 | 47.3 ± 7.5 | 62.7 ± 5.7 | 46.3 ± 0.2 | 63.8 ± 6.0 |
|  | U23 | 44.4 | 66.6 ± 5.1 | 55.2 ± 3.1 | 63.8 ± 3.7 |
| **VO_2max_**  **(mL·min^−1^)** | U16 | 3279 ± 719 | 4299 ± 996 | 3014 ± 308 | 4126 ± 1013 |
|  | U18 | 3638 ± 328 | 4776 ± 684 | 3134 ± 337 | 4263 ± 579 |
|  | U20 | 3409 ± 225 | 5229 ± 264 | 3471 ± 469 | 5433 ± 457 |
|  | U23 | 3559 | 5580 ± 196 | 3922 ± 350 | 5724 ± 275 |
| **VO_2max_·BM^−1^**  **(mL·kg^−1^· min^−1^)** | U16 | 61.1 ± 1.7 | 70.8 ± 6.2 | 59.2 ± 3.3 | 63.9 ± 19.5 |
|  | U18 | 60.5 ± 5.1 | 71.1 ± 6.5 | 60.7 ± 5.1 | 67.9 ± 7.6 |
|  | U20 | 54.9 ± 4.5 | 69.1 ± 4.0 | 61.3 ± 2.5 | 76.1 ± 8.0 |
|  | U23 | 52.6 | 76.2 ± 5.4 | 63.5 ± 3.3 | 75.8 ± 3.1 |

## 2.2 Sensitivity Analysis

To examine the robustness of the main findings, sensitivity analyses were conducted using separate univariate general linear models within an ANCOVA framework for all dependent variables. The primary model (M_0_) included age group and sex as fixed factors and their interaction. Sensitivity models were then specified to evaluate the extent to which structural factors related to training background and competitive level (M1: sport discipline and competitive level) and anthropometric characteristics (M2: body height; M3: body mass) attenuated age- and sex-related effects. For all models, η²ₚ values were calculated for each term and compared with the corresponding effect sizes from the primary model (M_0_). No additional post-hoc analyses were performed for the sensitivity models, as their only purpose was to assess the stability of effect sizes rather than to generate new inferential comparisons.

In the sensitivity analyses, changes in η²ₚ across models M_1_-M_3_ were compared with the corresponding effect sizes obtained from the primary model (M_0_) to assess whether age- or sex-related effects were influenced by structural (sport discipline or competitive level) or anthropometric characteristics (body height and body mass). A substantial reduction in η²ₚ after inclusion of a given covariate was interpreted as indicating that this factor accounted for part of the observed group differences. Conversely, stable effect sizes across all models were interpreted as evidence that age- and sex-related effects were robust and not driven by confounding structural or anthropometric influences.

| model | variable | term | η²ₚ | η²ₚ (M0) | Δ η²ₚ |
| --- | --- | --- | --- | --- | --- |
| M1 (Structure) | body height | age group | 0.44 | 0.43 | 0.00 |
|  |  | sex | 0.66 | 0.66 | 0.00 |
|  |  | sport discipline | 0.01 |  |  |
|  |  | competitive level | 0.01 |  |  |
|  |  | age group:sex | 0.07 | 0.06 | 0.00 |
| M2 (body height) | body height | age group | 0.43 | 0.43 | 0.00 |
|  |  | sex | 0.66 | 0.66 | 0.00 |
|  |  | age group:sex | 0.06 | 0.06 | 0.00 |
| M3 (body mass) | body height | age group | 0.59 | 0.43 | 0.16 |
|  |  | sex | 0.79 | 0.66 | 0.13 |
|  |  | body mass | 0.49 |  |  |
|  |  | age group:sex | 0.08 | 0.06 | 0.02 |
| M1 (Structure) | body mass | age group | 0.52 | 0.49 | 0.03 |
|  |  | sex | 0.41 | 0.39 | 0.02 |
|  |  | sport discipline | 0.09 |  |  |
|  |  | competitive level | 0.01 |  |  |
|  |  | age group:sex | 0.05 | 0.04 | 0.01 |
| M2 (body height) | body mass | age group | 0.65 | 0.49 | 0.16 |
|  |  | sex | 0.55 | 0.39 | 0.16 |
|  |  | body height | 0.48 |  |  |
|  |  | age group:sex | 0.06 | 0.04 | 0.02 |
| M3 (body mass) | body mass | age group | 0.49 | 0.49 | 0.00 |
|  |  | sex | 0.39 | 0.39 | 0.00 |
|  |  | age group:sex | 0.04 | 0.04 | 0.00 |
| M1 (Structure) | 1RM_pull_ | age group | 0.75 | 0.72 | 0.03 |
|  |  | sex | 0.71 | 0.68 | 0.03 |
|  |  | sport discipline | 0.12 |  |  |
|  |  | competitive level | 0.01 |  |  |
|  |  | age group:sex | 0.14 | 0.13 | 0.02 |
| M2 (body height) | 1RM_pull_ | age group | 0.73 | 0.72 | 0.00 |
|  |  | sex | 0.69 | 0.68 | 0.00 |
|  |  | body height | 0.03 |  |  |
|  |  | age group:sex | 0.12 | 0.13 | -0.01 |
| M3 (body mass) | 1RM_pull_ | age group | 0.79 | 0.72 | 0.06 |
|  |  | sex | 0.75 | 0.68 | 0.07 |
|  |  | body mass | 0.34 |  |  |
|  |  | age group:sex | 0.09 | 0.13 | -0.04 |
| M1 (Structure) | 1RM_press_ | age group | 0.70 | 0.67 | 0.04 |
|  |  | sex | 0.72 | 0.69 | 0.04 |
|  |  | sport discipline | 0.13 |  |  |
|  |  | competitive level | 0.04 |  |  |
|  |  | age group:sex | 0.14 | 0.12 | 0.02 |
| M2 (body height) | 1RM_press_ | age group | 0.67 | 0.67 | 0.00 |
|  |  | sex | 0.69 | 0.69 | 0.00 |
|  |  | body height | 0.01 |  |  |
|  |  | age group:sex | 0.12 | 0.12 | 0.00 |
| M3 (body mass) | 1RM_press_ | age group | 0.71 | 0.67 | 0.05 |
|  |  | sex | 0.73 | 0.69 | 0.05 |
|  |  | body mass | 0.25 |  |  |
|  |  | age group:sex | 0.09 | 0.12 | -0.03 |
| M1 (Structure) | 1RM_pull_·BM^-1^ | age group | 0.50 | 0.49 | 0.01 |
|  |  | sex | 0.50 | 0.49 | 0.01 |
|  |  | sport discipline | 0.03 |  |  |
|  |  | competitive level | 0.00 |  |  |
|  |  | age group:sex | 0.02 | 0.02 | 0.00 |
| M2 (body height) | 1RM_pull_·BM^-1^ | age group | 0.55 | 0.49 | 0.05 |
|  |  | sex | 0.54 | 0.49 | 0.05 |
|  |  | body height | 0.17 |  |  |
|  |  | age group:sex | 0.05 | 0.02 | 0.03 |
| M3 (body mass) | 1RM_pull_·BM^-1^ | age group | 0.51 | 0.49 | 0.02 |
|  |  | sex | 0.51 | 0.49 | 0.02 |
|  |  | body mass | 0.06 |  |  |
|  |  | age group:sex | 0.04 | 0.02 | 0.02 |
| M1 (Structure) | 1RM_push_·BM^-1^ | age group | 0.49 | 0.47 | 0.02 |
|  |  | sex | 0.58 | 0.56 | 0.02 |
|  |  | sport discipline | 0.04 |  |  |
|  |  | competitive level | 0.03 |  |  |
|  |  | age group:sex | 0.04 | 0.03 | 0.01 |
| M2 (body height) | 1RM_push_·BM^-1^ | age group | 0.50 | 0.47 | 0.04 |
|  |  | sex | 0.59 | 0.56 | 0.04 |
|  |  | body height | 0.12 |  |  |
|  |  | age group:sex | 0.05 | 0.03 | 0.02 |
| M3 (body mass) | 1RM_push_·BM^-1^ | age group | 0.48 | 0.47 | 0.01 |
|  |  | sex | 0.57 | 0.56 | 0.01 |
|  |  | body mass | 0.02 |  |  |
|  |  | age group:sex | 0.04 | 0.03 | 0.01 |
| M1 (Structure) | DPP_15s_ | age group | 0.63 | 0.60 | 0.02 |
|  |  | sex | 0.64 | 0.62 | 0.02 |
|  |  | sport discipline | 0.06 |  |  |
|  |  | competitive level | 0.04 |  |  |
|  |  | age group:sex | 0.05 | 0.05 | 0.00 |
| M2 (body height) | DPP_15s_ | age group | 0.62 | 0.60 | 0.02 |
|  |  | sex | 0.63 | 0.62 | 0.02 |
|  |  | body height | 0.07 |  |  |
|  |  | age group:sex | 0.04 | 0.05 | -0.01 |
| M3 (body mass) | DPP_15s_ | age group | 0.67 | 0.60 | 0.07 |
|  |  | sex | 0.69 | 0.62 | 0.07 |
|  |  | body mass | 0.28 |  |  |
|  |  | age group:sex | 0.02 | 0.05 | -0.03 |
| M1 (Structure) | DPP_peak_ | age group | 0.73 | 0.67 | 0.07 |
|  |  | sex | 0.75 | 0.69 | 0.06 |
|  |  | sport discipline | 0.16 |  |  |
|  |  | competitive level | 0.21 |  |  |
|  |  | age group:sex | 0.13 | 0.14 | -0.01 |
| M2 (body height) | DPP_peak_ | age group | 0.70 | 0.67 | 0.03 |
|  |  | sex | 0.72 | 0.69 | 0.03 |
|  |  | body height | 0.18 |  |  |
|  |  | age group:sex | 0.13 | 0.14 | -0.01 |
| M3 (body mass) | DPP_peak_ | age group | 0.76 | 0.67 | 0.10 |
|  |  | sex | 0.78 | 0.69 | 0.09 |
|  |  | body mass | 0.43 |  |  |
|  |  | age group:sex | 0.11 | 0.14 | -0.03 |
| M1 (Structure) | DPP_15s_·BM^-1^ | age group | 0.50 | 0.49 | 0.01 |
|  |  | sex | 0.56 | 0.55 | 0.01 |
|  |  | sport discipline | 0.01 |  |  |
|  |  | competitive level | 0.04 |  |  |
|  |  | age group:sex | 0.01 | 0.00 | 0.00 |
| M2 (body height) | DPP_15s_·BM^-1^ | age group | 0.49 | 0.49 | 0.00 |
|  |  | sex | 0.55 | 0.55 | 0.00 |
|  |  | body height | 0.01 |  |  |
|  |  | age group:sex | 0.01 | 0.00 | 0.00 |
| M3 (body mass) | DPP_15s_·BM^-1^ | age group | 0.49 | 0.49 | 0.00 |
|  |  | sex | 0.55 | 0.55 | 0.00 |
|  |  | body mass | 0.00 |  |  |
|  |  | age group:sex | 0.00 | 0.00 | 0.00 |
| M1 (Structure) | DPP_peak_·BM^-1^ | age group | 0.57 | 0.50 | 0.07 |
|  |  | sex | 0.67 | 0.61 | 0.07 |
|  |  | sport discipline | 0.08 |  |  |
|  |  | competitive level | 0.23 |  |  |
|  |  | age group:sex | 0.03 | 0.06 | -0.03 |
| M2 (body height) | DPP_peak_·BM^-1^ | age group | 0.50 | 0.50 | 0.00 |
|  |  | sex | 0.61 | 0.61 | 0.00 |
|  |  | body height | 0.00 |  |  |
|  |  | age group:sex | 0.07 | 0.06 | 0.01 |
| M3 (body mass) | DPP_peak_·BM^-1^ | age group | 0.50 | 0.50 | 0.00 |
|  |  | sex | 0.61 | 0.61 | 0.00 |
|  |  | body mass | 0.00 |  |  |
|  |  | age group:sex | 0.07 | 0.06 | 0.00 |
| M1 (Structure) | VO_2peak_ DP | age group | 0.62 | 0.57 | 0.05 |
|  |  | sex | 0.70 | 0.66 | 0.04 |
|  |  | sport discipline | 0.08 |  |  |
|  |  | competitive level | 0.17 |  |  |
|  |  | age group:sex | 0.11 | 0.14 | -0.03 |
| M2 (body height) | VO_2peak_ DP | age group | 0.63 | 0.57 | 0.05 |
|  |  | sex | 0.71 | 0.66 | 0.05 |
|  |  | body height | 0.24 |  |  |
|  |  | age group:sex | 0.13 | 0.14 | -0.01 |
| M3 (body mass) | VO_2peak_ DP | age group | 0.74 | 0.57 | 0.16 |
|  |  | sex | 0.80 | 0.66 | 0.14 |
|  |  | body mass | 0.56 |  |  |
|  |  | age group:sex | 0.12 | 0.14 | -0.02 |
| M1 (Structure) | VO_2max_ | age group | 0.62 | 0.60 | 0.03 |
|  |  | sex | 0.73 | 0.70 | 0.02 |
|  |  | sport discipline | 0.03 |  |  |
|  |  | competitive level | 0.12 |  |  |
|  |  | age group:sex | 0.09 | 0.12 | -0.03 |
| M2 (body height) | VO_2max_ | age group | 0.66 | 0.60 | 0.07 |
|  |  | sex | 0.76 | 0.70 | 0.06 |
|  |  | body height | 0.27 |  |  |
|  |  | age group:sex | 0.12 | 0.12 | 0.00 |
| M3 (body mass) | VO_2max_ | age group | 0.74 | 0.60 | 0.15 |
|  |  | sex | 0.82 | 0.70 | 0.12 |
|  |  | body mass | 0.53 |  |  |
|  |  | age group:sex | 0.09 | 0.12 | -0.03 |
| M1 (Structure) | VO_2peak_ DP·BM^-1^ | age group | 0.26 | 0.21 | 0.05 |
|  |  | sex | 0.58 | 0.51 | 0.07 |
|  |  | sport discipline | 0.03 |  |  |
|  |  | competitive level | 0.28 |  |  |
|  |  | age group:sex | 0.06 | 0.11 | -0.05 |
| M2 (body height) | VO_2peak_ DP·BM^-1^ | age group | 0.21 | 0.21 | 0.00 |
|  |  | sex | 0.51 | 0.51 | 0.00 |
|  |  | body height | 0.00 |  |  |
|  |  | age group:sex | 0.10 | 0.11 | 0.00 |
| M3 (body mass) | VO_2peak_ DP·BM^-1^ | age group | 0.21 | 0.21 | 0.00 |
|  |  | sex | 0.51 | 0.51 | 0.00 |
|  |  | body mass | 0.01 |  |  |
|  |  | age group:sex | 0.10 | 0.11 | 0.00 |
| M1 (Structure) | VO_2max_·BM^-1^ | age group | 0.19 | 0.18 | 0.02 |
|  |  | sex | 0.52 | 0.49 | 0.03 |
|  |  | sport discipline | 0.01 |  |  |
|  |  | competitive level | 0.13 |  |  |
|  |  | age group:sex | 0.03 | 0.05 | -0.02 |
| M2 (body height) | VO_2max_·BM^-1^ | age group | 0.18 | 0.18 | 0.00 |
|  |  | sex | 0.49 | 0.49 | 0.00 |
|  |  | body height | 0.01 |  |  |
|  |  | age group:sex | 0.06 | 0.05 | 0.01 |
| M3 (body mass) | VO_2max_·BM^-1^ | age group | 0.18 | 0.18 | 0.00 |
|  |  | sex | 0.49 | 0.49 | 0.01 |
|  |  | body mass | 0.02 |  |  |
|  |  | age group:sex | 0.06 | 0.05 | 0.01 |

## 2.3 Supplementary Figures

Figure 1 Distribution of body mass normalized maximal strength, power output during DP ergometry, and aerobic power across age groups and sex. Box plots represent the median and interquartile range, with whiskers indicating the data range. Individual data points are overlaid as scatter plots to illustrate inter-individual variability. Diamonds indicate group means. Point color denotes performance level, and point shape indicates sport discipline. Abbreviations: 1RM, one‐repetition maximum; DPP_15s_, mean power during 15s double poling ergometer sprint; DPP_PEAK_, peak power during the double poling ergometry incremental test to exhaustion; VO_2max_, maximal oxygen uptake; VO_2peak_ DP, peak oxygen uptake during double poling ergometry; BM, body mass.

Figure 2 Pairwise scatterplot matrix illustrating the relationships between maximal strength, DP performance, and aerobic power. All variables were residualized for sex and age and subsequently z-standardized for graphical presentation. The lower triangle displays scatterplots of sex-adjusted residuals, with individual observations color-coded by sex (male = blue, female = red). Linear regression lines (black) with 95% confidence intervals (grey). Diagonal panels depict kernel density estimates of the standardized residuals. The upper triangle presents partial Pearson correlation coefficients (r), controlling for sex and age, visualized as heatmap-style tiles (blue = negative, red = positive); asterisks indicate statistically significant correlations (p < 0.05). Abbreviations: 1RM, one‐repetition maximum; DPP_15s_, mean power during 15s double poling ergometer sprint; DPP_PEAK_, peak power during the double poling ergometry incremental test to exhaustion; VO_2max_, maximal oxygen uptake; VO_2peak_ DP, peak oxygen uptake during double poling ergometry; BM, body mass.

Figure 3 Heatmap illustrating Pearson correlation coefficient (r) between maximal strength, power output during double poling ergometry, and aerobic power. Correlation magnitudes are interpreted as ≤0.1=trivial, >0.1–0.3=small,>0.3–0.5=moderate, and>0.5=large, with these thresholds visually encoded by the color scale. Statistical significance is denoted by asterisks (* p<0.05, **p<0.01, ***p<0.001). Abbreviations: 1RM, one‐repetition maximum; DPP_15s_, mean power during 15s double poling ergometer sprint; DPP_PEAK_, peak power during the double poling ergometry incremental test to exhaustion; VO_2max_, maximal oxygen uptake; VO_2peak_ DP, peak oxygen uptake during double poling ergometry.

Figure 4 Heatmap illustrating Pearson correlation coefficient (r) between maximal strength, power output during double poling ergometry, and aerobic power. Correlation magnitudes are interpreted as ≤0.1=trivial, >0.1–0.3=small,>0.3–0.5=moderate, and>0.5=large, with these thresholds visually encoded by the color scale. Statistical significance is denoted by asterisks (* p<0.05, **p<0.01, ***p<0.001). Abbreviations: 1RM, one‐repetition maximum; DPP_15s_, mean power during 15s double poling ergometer sprint; DPP_PEAK_, peak power during the double poling ergometry incremental test to exhaustion; VO_2max_, maximal oxygen uptake; VO_2peak_ DP, peak oxygen uptake during double poling ergometry; BM, body mass.

**Figure** 5 Scatterplots showing Pearson correlations between maximal strength and peak power during ramp test to exhaustion. Blue points represent male athletes; red points represent female athletes. The black line indicates the regression slope, illustrating the direction and strength of the relationship. Correlation coefficients (r-values) are reported. Abbreviations: 1RM, one‐repetition maximum; DPP_PEAK_, peak power during the double poling ergometry incremental test to exhaustion; BM, body mass.

**Figure 6** Scatterplots showing Pearson correlations between maximal strength and mean power during 15s double poling ergometer sprint. Blue points represent male athletes; red points represent female athletes. The black line indicates the regression slope, illustrating the direction and strength of the relationship. Correlation coefficients (r-values) are reported. Abbreviations: 1RM, one‐repetition maximum; DPP_15s_, mean power during 15s double poling ergometer sprint; BM, body mass.

Figure 7 Scatterplots showing Pearson correlations between maximal strength and peak oxygen uptake during double poling ergometry. Blue points represent male athletes; red points represent female athletes. The black line indicates the regression slope, illustrating the direction and strength of the relationship. Correlation coefficients (r-values) are reported. Abbreviations: 1RM, one‐repetition maximum; VO_2peak_ DP, peak oxygen uptake during double poling ergometry; BM, body mass.

Figure 8 Scatterplots showing Pearson correlations between peak oxygen uptake during double poling ergometry and power output during double poling ergometry. Blue points represent male athletes; red points represent female athletes. The black line indicates the regression slope, illustrating the direction and strength of the relationship. Correlation coefficients (r-values) are reported. Abbreviations: DPP_15s_, mean power during 15s double poling ergometer sprint; DPP_PEAK_, peak power during the double poling ergometry incremental test to exhaustion; VO_2peak_ DP, peak oxygen uptake during double poling ergometry; BM, body mass.

Figure 9 Scatterplots showing Pearson correlations between maximal oxygen and power output during double poling ergometry. Blue points represent male athletes; red points represent female athletes. The black line indicates the regression slope, illustrating the direction and strength of the relationship. Correlation coefficients (r-values) are reported. Abbreviations: DPP_15s_, mean power during 15s double poling ergometer sprint; DPP_PEAK_, peak power during the double poling ergometry incremental test to exhaustion; VO_2max_, maximal oxygen uptake; BM, body mass.

Figure 10 Heatmap illustrating partial Pearson correlation coefficient (r) between maximal strength, power output during double poling ergometry, and aerobic power, controlling for age and sex. Correlation magnitudes are interpreted as ≤0.1=trivial, >0.1–0.3=small,>0.3–0.5=moderate, and>0.5=large, with these thresholds visually encoded by the color scale. Statistical significance is denoted by asterisks (* p<0.05, **p<0.01, ***p<0.001). Abbreviations: 1RM, one‐repetition maximum; DPP_15s_, mean power during 15s double poling ergometer sprint; DPP_PEAK_, peak power during the double poling ergometry incremental test to exhaustion; VO_2max_, maximal oxygen uptake; VO_2peak_ DP, peak oxygen uptake during double poling ergometry.

Figure 11 Heatmap illustrating partial Pearson correlation coefficient (r) between maximal strength, power output during double poling ergometry, and aerobic power, controlling for age and sex. Correlation magnitudes are interpreted as ≤0.1=trivial, >0.1–0.3=small,>0.3–0.5=moderate, and>0.5=large, with these thresholds visually encoded by the color scale. Statistical significance is denoted by asterisks (* p<0.05, **p<0.01, ***p<0.001). Abbreviations: 1RM, one‐repetition maximum; DPP_15s_, mean power during 15s double poling ergometer sprint; DPP_PEAK_, peak power during the double poling ergometry incremental test to exhaustion; VO_2max_, maximal oxygen uptake; VO_2peak_ DP, peak oxygen uptake during double poling ergometry; BM, body mass.

Figure 12 Scatterplots showing partial Pearson correlations between maximal strength and peak power during ramp test to exhaustion, controlling for age and sex. Blue points represent male athletes; red points represent female athletes. The black line indicates the regression slope, illustrating the direction and strength of the relationship. Partial correlation coefficients (r-values) are reported. Abbreviations: 1RM, one‐repetition maximum; DPP_PEAK_, peak power during the double poling ergometry incremental test to exhaustion; BM, body mass.

Figure 13 Scatterplots showing partial Pearson correlations between maximal strength and mean power during sprint test, controlling for age and sex. Blue points represent male athletes; red points represent female athletes. The black line indicates the regression slope, illustrating the direction and strength of the relationship. Partial correlation coefficients (r-values) are reported. Abbreviations: 1RM, one‐repetition maximum; DPP_15s_, mean power during 15s double poling ergometer sprint; BM, body mass.

Figure 14 Scatterplots showing partial Pearson correlations between maximal strength and peak oxygen uptake during DP, controlling for age and sex. Blue points represent male athletes; red points represent female athletes. The black line indicates the regression slope, illustrating the direction and strength of the relationship. Partial correlation coefficients (r-values) are reported. Abbreviations: 1RM, one‐repetition maximum; VO_2peak_ DP, peak oxygen uptake during double poling ergometry; BM, body mass.

Figure 15 Scatterplots showing partial Pearson correlations between peak oxygen uptake during DP and power output during double poling ergometry, controlling for age and sex. Blue points represent male athletes; red points represent female athletes. The black line indicates the regression slope, illustrating the direction and strength of the relationship. Partial correlation coefficients (r-values) are reported. Abbreviations: DPP_15s_, mean power during 15s double poling ergometer sprint; DPP_PEAK_, peak power during the double poling ergometry incremental test to exhaustion; VO_2peak_ DP, peak oxygen uptake during double poling ergometry; BM, body mass.

Figure 16 Scatterplots showing partial Pearson correlations between between maximal oxygen and power output during double poling ergometry, controlling for age and sex. Blue points represent male athletes; red points represent female athletes. The black line indicates the regression slope, illustrating the direction and strength of the relationship. Partial correlation coefficients (r-values) are reported. Abbreviations: DPP_15s_, mean power during 15s double poling ergometer sprint; DPP_PEAK_, peak power during the double poling ergometry incremental test to exhaustion; VO_2max_, maximal oxygen uptake; BM, body mass.
